# Predicting Interfacial Pull-Out Performance of Nano-B_4_C/Aramid Material with Stage-Wise Physics-Guided Machine Learning

**DOI:** 10.3390/polym18131618

**Published:** 2026-06-29

**Authors:** Havva Esra Bakbak, Aytuğ Onan, Erman Bilisik, Kadir Bilisik

**Affiliations:** 1 Department of Electrical and Electronic Engineering, Ege University, Izmir 35040, Turkey; bilisik.h@gmail.com; 2 Department of Computer Engineering, Izmir Institute of Technology, Izmir 35430, Turkey; aytug.onan@ikc.edu.tr; 3 Nanotechnology Application and Research Centre (ERNAM), Erciyes University, Kayseri 38039, Turkey; ermanbilisik66@gmail.com; 4 Department of Nanoscience and Nanotechnology, Graduate School of Natural and Applied Sciences, Erciyes University, Kayseri 38039, Turkey; 5 Nano/Microfiber Preform Design and Composite Laboratory, Department of Textile Engineering, Faculty of Engineering, Erciyes University, Kayseri 38039, Turkey

**Keywords:** aramid fiber, nano hexagonal boron carbide (nh-B4C), pull-out force, shear, machine learning

## Abstract

Interfacial yarn pull-out plays a critical role in load transfer and energy dissipation in soft ballistic materials; however, its multistage and friction-dominated nature makes comprehensive experimental characterization time-consuming and experimentally demanding. In this study, a stage-wise physics-guided machine learning (PG-HML) framework is proposed to predict the pull-out behavior of nano hexagonal boron carbide (nh-B_4_C)-functionalized para-aramid fabrics using an experimentally constrained dataset. The pull-out response was decomposed into three physically meaningful deformation stages, namely crimp extension, initial interlacement rupture, and stick–slip sliding. Physics-based continuity and admissibility constraints were incorporated into the learning framework to preserve mechanical consistency across stage transitions and improve prediction robustness under limited data conditions. Comparative analyses demonstrated that the proposed PG-HML framework achieved superior predictive capability, particularly in capturing rupture transitions and post-peak stick–slip evolution, with R^2^ values exceeding 0.98 during the crimp extension and rupture stages. Increasing nh-B_4_C content enhanced interfacial friction, rupture resistance, and pull-out energy dissipation, while displacement responses gradually approached saturation under force-dominated extraction conditions. Therefore, interfacial pull-out behavior in nh-B_4_C/aramid materials can be predicted with high fidelity using limited experimental input, providing a surrogate modeling strategy that enables virtual material screening and significantly reduces the experimental effort required for preliminary design and optimization of advanced soft ballistic materials.

## 1. Introduction

Machine learning (ML), a central branch of artificial intelligence, has evolved into a powerful data-driven framework for identifying patterns, learning complex relationships, and generating predictive models directly from experimental or numerical data without explicit rule-based programming [1]. Unlike conventional artificial intelligence (AI) approaches relying on predefined logical structures, ML algorithms infer governing trends from data, enabling robust prediction and decision-making across scientific and engineering applications. Among major ML paradigms, supervised learning has become the most widely used approach for regression and classification tasks in materials research [2,3]. Its increasing adoption in material engineering is primarily driven by its capability to generalize learned relationships to unseen conditions, particularly where experimental testing, high-fidelity simulations, or multiscale modeling are costly or impractical [4,5,6,7,8]. As a result, ML-based surrogate models are increasingly regarded as scalable and computationally efficient alternatives to traditional empirical design methods and physics-based multiscale approaches [9,10,11], positioning ML as a key enabler for accelerated materials discovery, characterization, and optimization. Recently, increasing attention has been devoted to physics-guided and physics-informed machine learning frameworks, which integrate experimentally or theoretically established physical knowledge with data-driven learning algorithms. By embedding physical constraints, governing mechanisms, or domain-specific knowledge into the learning process, these approaches improve model interpretability, generalization capability, and robustness, particularly when only limited experimental datasets are available [12,13,14]. The proposed PG-HML framework follows this general philosophy by incorporating experimentally established pull-out deformation mechanisms into a stage-wise supervised learning framework.

Building on its success in broader materials science, ML has recently been extended to textile materials, where hierarchical architectures and strong process–structure–property coupling challenge conventional modeling approaches. ML and deep learning techniques have been applied to fiber identification, fabric classification, and tactile property assessment, including fabric hand and softness, primarily through image-based learning frameworks. These methods have shown promising predictive performance, particularly where expert-driven or experimental evaluations are limited by subjectivity, efficiency, or scalability. Nevertheless, challenges related to model generalization, robust feature representation, and industrial transferability persist, highlighting the need for application-oriented and transferable ML frameworks tailored to fiber systems [15,16,17,18]. Beyond image-based characterization, ML also provides significant potential for modeling mechanically driven textile phenomena governed by frictional and interfacial interactions. Among these, yarn pull-out constitutes a primary energy dissipation mechanism in protective fiber-based structures and plays a critical role in ballistic and impact resistance [19]. The pull-out response is mainly governed by inter-yarn friction, yarn crimp, fabric architecture, and extraction configuration.

Significant enhancements in ballistic and stab resistance have been achieved using high-performance para-aramid fibers, optimized matrix systems, and advanced two- and three-dimensional fabric architectures [20]. To further strengthen interfacial interactions, various surface modification strategies have been explored, including chemical treatments [21], nano-coatings [22], polymer grafting [23], and in situ nanostructure growth [24], all aimed at improving mechanical interlocking and chemical adhesion at fiber interfaces [25]. More recently, laser surface texturing has emerged as an effective and controllable approach for tailoring surface morphology in textile materials. In para-aramid fabrics, laser-induced roughness has been shown to enhance yarn–yarn interactions and increase pull-out resistance, leading to improved impact energy absorption [26]. Likewise, studies on Kevlar^®^ fabrics report that metallic surface coatings significantly increase peak pull-out force and absorbed energy by amplifying inter-yarn friction through controlled roughness modification [27].

In parallel with these developments, the incorporation of nanoscale reinforcements, including carbon nanotubes (CNTs) [28], graphene nanoplatelets (GNPs) [29,30], nano-structured h-B_4_C [31,32,33,34], shear thickening fluids (STFs) [35,36], and electrospun nanofibers [37,38,39], has enabled the development of lightweight ballistic composites with enhanced energy absorption and damage tolerance [40,41,42,43,44,45]. STFs are widely employed to improve yarn pull-out resistance by increasing inter-yarn friction and impact energy dissipation [46,47], while multi-phase STFs containing additives such as silicon carbide (SiC) and CNTs exhibit stronger thickening responses, reducing yarn slippage and improving load transfer under quasi-static and dynamic loading [48,49,50]. Similarly, surface modification approaches using zinc oxide (ZnO) nanowires and silicon dioxide (SiO_2_) treatments have demonstrated increased inter-fiber friction and strain energy dissipation in aramid fabrics, particularly at elevated strain rates [51]. Beyond fluid-based systems, advances in aramid nanofibers (ANFs), CNT-grafted aramids, and chemically modified fiber surfaces have enabled substantial improvements in fiber–matrix adhesion and interfacial strength [52,53,54,55,56]. ANF-engineered interphases, in particular, have yielded significant enhancements in the tensile, flexural, interlaminar shear, and impact performance of aramid-reinforced composites [57,58]. Collectively, these nano-engineering strategies underscore the strong sensitivity of pull-out behavior to multiscale design parameters, providing a robust foundation for machine learning-assisted modeling and optimization using nanoscale, interfacial, and processing descriptors.

Yarn pull-out is a dominant energy dissipation mechanism in p-aramid fabrics under ballistic and impact loading, governed by yarn uncrimping followed by translational motion [59,60]. The pull-out response of high-performance woven fabrics is strongly affected by interlacement pattern, pull-out rate, inter-yarn friction, areal density, specimen geometry, and the number of extracted yarns. Multi-yarn configurations typically exhibit higher resistance than single-yarn cases due to cumulative friction and load-sharing effects [61]. Increased pull-out force is directly linked to improved impact performance and can be tuned through yarn linear density, crimp level, and inter-yarn friction, whereas moisture-induced lubrication reduces frictional resistance and contracts the pull-out interaction zone [62,63]. Stick–slip behavior and cumulative retraction forces intensify with increasing fabric density and yarn count, while higher pull-out velocities tend to lower force levels in the stick–slip regime due to reduced interaction time at yarn crossover points [64,65]. Despite extensive experimental efforts, the interpretation of stick–slip signals, particularly at small length scales, remains largely qualitative and case-dependent [66]. Recent studies demonstrate that machine learning, especially neural network-based frameworks, can extract physically meaningful parameters from frictional force–displacement responses, enabling automated, robust, and physics-consistent characterization of stick–slip behavior even under data-scarce conditions [67]. This capability highlights the potential of ML-assisted approaches to advance multiscale pull-out analysis while reducing the experimental burden.

ML has become a powerful modeling and optimization tool for composite materials, offering substantial reductions in computational cost and experimental effort compared to conventional trial-and-error or high-fidelity numerical approaches. Once adequately trained, ML algorithms can accurately predict the mechanical properties, constitutive responses, and failure-related parameters of composite systems [68,69,70,71]. ML-assisted multiscale strategies have successfully replaced micromechanical finite element analyses through neural network-based surrogate models, enabling efficient simulation of woven and laminated composites [72]. Integration of ML with continuum damage mechanics has further enabled the direct identification of damage parameters from macroscopic responses, reducing dependence on destructive testing and complex non-destructive evaluation methods [73]. At smaller length scales, ML frameworks coupled with molecular dynamics simulations have been proposed to predict composite behavior from resin- and interface-level descriptors [74], while recent reviews emphasize the critical role of MD-driven nano-mechanics in understanding interfacial degradation and durability [75]. Moreover, advances in multimodal and hybrid learning architectures combining convolutional neural networks with multilayer perceptrons have improved predictive accuracy by jointly incorporating compositional and microstructural features, addressing the limitations of single-source learning and enhancing the robustness of structure–property relationships [76].

Accurate prediction of ballistic and impact responses in textile-based armor systems is critical for designing lightweight protective structures, yet conventional approaches rely heavily on costly and destructive experimental testing. Recent studies show that ML models can effectively predict key ballistic performance metrics, including penetration depth, residual velocity, and energy dissipation, using intrinsic material and structural parameters, providing efficient alternatives to traditional analytical formulations [77]. ML-guided optimization has further enabled the design of hybrid protective systems by identifying governing variables, such as yarn friction coefficient, tensile strength, and transverse wave velocity, that control impact resistance [78]. At the system level, ML models have also been applied to multilayer ceramic–composite armor configurations, accurately predicting residual projectile velocity and force–displacement responses while substantially reducing computational costs [79,80,81].

Despite extensive experimental and numerical studies on p-aramid fabrics, machine learning-based investigations of yarn pull-out behavior, particularly under single- and multi-yarn extraction, remain limited. Existing approaches rely on either extensive experimentation or computationally intensive physics-based models, restricting efficient exploration of nano-functionalization effects. In particular, the role of nano-structured hexagonal boron carbide (nh-B_4_C) in governing interfacial mechanics and energy dissipation during pull-out has not been quantitatively addressed within ML-assisted frameworks. To address this gap, this study proposes a data-efficient machine learning methodology to predict the pull-out performance of nano-functionalized p-aramid fabrics across varying nh-B_4_C contents, enabling reliable force and energy estimation while significantly reducing experimental effort.

The central objective of this work is to develop and validate a stage-wise physics-guided hybrid machine learning (PG-HML) framework for predicting and optimizing the pull-out behavior of nano-reinforced soft ballistic materials under data-scarce conditions commonly encountered in experimental textile mechanics. The scientific novelty of the proposed methodology lies in the integration of physically meaningful deformation-stage decomposition, supervised regression learning, and physics-informed continuity constraints within a unified predictive framework. Unlike conventional black-box machine learning approaches, the pull-out response is separated into three distinct mechanical stages—crimp extension, initial interlacement rupture, and stick–slip sliding—thereby preserving mechanical interpretability while enabling stage-specific deformation mechanisms to be identified and quantified. Furthermore, the incorporation of physics-guided constraints ensures mechanically admissible transitions between deformation regions and reduces the likelihood of non-physical predictions. The framework operates effectively using a limited experimental dataset, addressing practical situations in which generating large training datasets is impractical due to material cost, testing complexity, and experimental time requirements. By integrating interfacial mechanics, nano-functionalization effects, and data-driven modeling, the proposed PG-HML framework enables virtual exploration of nano-additive concentrations prior to physical testing and provides an efficient tool for the design, optimization, and performance assessment of next-generation soft ballistic systems. Accordingly, the proposed PG-HML framework enables virtual exploration of nano-additive concentrations and associated pull-out parameters before additional experimental testing, thereby reducing the experimental effort required during material development and preliminary optimization.

## 2. Materials and Methods

### 2.1. Materials

The constituent para-aramid fibers were characterized by an average diameter of 12 μm, a density of 1.45 g/cm^3^, a tensile strength of 3200 MPa, a tensile modulus of 115 GPa, and an elongation at break of 2.9% [82]. The reinforcement fabric consisted of p-aramid yarns (Twaron^®^ CT 747, Teijin, Tokyo, Japan) arranged in a periodically ordered meso-scale architecture. A plain pattern was employed, in which both warp and weft yarns exhibited identical linear densities of 336 tex, resulting in a balanced and directionally symmetric yarn layout. The fabric exhibited an average yarn density of 6.25 ends/cm, a thickness of 0.62 mm, and an areal density of 410 g/m^2^. Crimp values in the warp and weft directions were comparable, ranging between 5.8 and 5.9%, and the fabric surface was finished with a water-repellent treatment. Nano-sized hexagonal boron carbide particles (nh-B_4_C, Nanografi, Türkiye (TR)) with a purity of 99.5%, a particle size distribution of 40–60 nm, a specific surface area of 55 m^2^/g, a tap density of 0.1 g/cm^3^, and a tensile modulus in the range of 0.35–0.55 Terapascal (TPa) were employed as the surface-modifying nanofiller for the p-aramid fabrics [83,84]. Ethanol (CH_3_CH_2_OH, 96% purity), used as the dispersion medium, was supplied by Rasel Kimya (United Ethanol Ind., Sadiqabad, Pakistan).

### 2.2. Fabric Preparation and Dataset Generation

The specifications of nanoparticle-coated pull-out specimens are summarized in Table 1. Two fabric types were considered in this study: a neat para-aramid fabric (KPO) and nano boron carbide-coated (BPO) aramid fabrics.

The fabrication procedure for the nh-B_4_C-coated para-aramid pull-out samples has been described in detail in earlier studies [85,86]; therefore, only a concise overview is provided here, with an emphasis on dataset generation for machine learning modeling. Aramid fabrics (120 mm × 300 mm) were first thermally conditioned at 60 °C to remove residual moisture and activate fiber surfaces. For BPO samples, nh-B_4_C nanoparticles (0.3 wt.%) were dispersed in ethanol using combined magnetic stirring and ultrasonication to obtain a homogeneous suspension. Fabrics were then immersed in the suspension and subjected to ultrasonic agitation to promote nanoparticle penetration, followed by a resting period under ambient conditions to enhance physicochemical interactions. An iterative immersion–compression method was subsequently applied, during which the fabrics were hot-pressed (2 bar, 65 °C) to promote nanoparticle infiltration into intra-filament gaps and yarn interlacement regions, thereby enhancing mechanical interlocking and interfacial adhesion [85]. This immersion–compression cycle was repeated three times to ensure uniform surface modification. Final drying was carried out at 78 °C. The resulting specimens were thus prepared with sufficient consistency for use in pull-out testing and subsequent machine learning analysis.

### 2.3. Analysis and Characterization

#### 2.3.1. Nano Pull-Out Testing and Energy Evaluation

Yarn pull-out tests were performed to quantify inter-yarn interlacement friction in plain (1/1) p-aramid fabrics under single-, double-, and triple-yarn extraction configurations using a custom-designed gripping fixture integrated with a universal testing machine (Instron 4411, 5 kN load cell, Instron Corporation, Norwood, MA, USA) [61]. Nano-coated fabric specimens with dimensions of 50 mm × 200 mm and a frayed edge length of 100 mm were tested along the warp direction under minimal pre-tension at a constant crosshead speed of 100 mm/min. Following single-yarn extraction, two- and three-yarn pull-out tests were conducted under identical testing conditions. The resulting force–displacement (F–δ) responses were recorded to identify the peak pull-out force, crimp extension behavior, initial interlacement rupture force corresponding to warp–weft disengagement, and characteristic stick–slip responses. Crimp extension was defined as the apparent yarn elongation arising from fabric interlacement effects [61], whereas pre-crimp fabric displacement and yarn slippage were neglected due to the imposed specimen geometry and constrained edge conditions. Pull-out energy absorption for single- and multi-yarn configurations was quantified from the area under the F–δ curves by separating the energy contributions associated with crimp extension, initial interlacement rupture, and stick–slip stages. The total absorbed energy was obtained by summing the stage-wise energy components. Pull-out energy was calculated by numerical integration of the force–displacement curves using the trapezoidal integration method, as defined by Equations (1)–(5), where Equation (1) represents the general energy formulation, and Equations (2), (3), and (4) correspond to the stage-wise energy expressions for Stage I, Stage II, and Stage III, respectively. All calculations were implemented using Python software (v3.1.2.4, Python Software Foundation, NL).

(1) $$E=\int F\left(x\right)dx\approx \sum_{i=1}^{N-1}\frac{F_{i}+F_{i+1}}{2}(x_{i+1}-x_{i})$$

(2) $$E_{I}=\int_{x_{0}}^{x_{max}}F\;dx$$

(3) $$E_{II}=\int_{x_{max}}^{x_{c}}F\;dx$$

(4) $$E_{III}=\int_{x_{c}}^{x_{end}}F\;dx$$

(5) $$E_{T}=E_{I}+E_{II}+E_{III}$$

where *E_I_* is the crimp extension energy (Stage I, J), *E_II_* is the initial interlacement rupture energy (Stage II, J), *E_III_* is the stick–slip energy (Stage III, J), and *E_T_* is the total energy (J).

#### 2.3.2. Intra-Yarn Shear Strength

Intra-yarn shear strength (IYSS) was evaluated to characterize shear deformation between interacting yarns during single- and multi-yarn pull-out along the warp direction, prior to the onset of stick–slip behavior. This parameter reflects the resistance associated with angular deformation of filling yarns within the fabric plane during pull-out loading. The IYSS of nano-coated fabrics was calculated using Equation (6):

(6) $$\tau_{iy}(IYSS)=\frac{F_{max}}{\pi \;R\;l_{y}}$$

where *τ*_*iy*_ denotes intra-yarn shear strength (Pa), *F*_max_ is the peak pull-out force (N), *R* is the yarn radius (mm), and *l*_*y*_ represents the effective yarn length engaged within the fabric (mm). Fabric crimp and wrinkle effects were neglected, and yarns were assumed to behave as inextensible multifilament assemblies [87]. All IYSS values were computed using Microsoft Excel (v2016).

#### 2.3.3. Physics-Guided Hybrid Machine Learning Framework for Pull-Out Modeling

**Figure 1:**
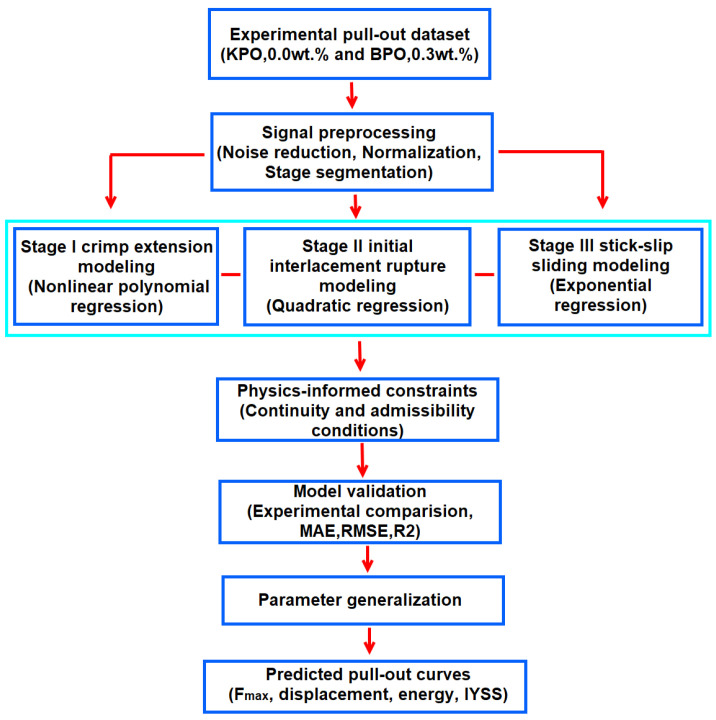
Schematic representation of the proposed stage-wise physics-guided hybrid machine learning (PG-HML) framework used to predict the single-, two-, and three-yarn pull-out force–displacement behavior of nh-B_4_C-functionalized para-aramid fabrics. The workflow integrates experimentally measured pull-out data, signal preprocessing, stage-wise regression modeling, physics-informed continuity constraints, model validation, and concentration-dependent parameter generalization to generate physically consistent predictions for untested nano-additive contents.

A physics-guided hybrid machine learning (PG-HML) framework was developed to predict the pull-out force–displacement behavior of nano-functionalized aramid fabrics for untested nh-B_4_C contents (0.1–1.0 wt.%). The present framework was developed following the general concepts of physics-guided (or physics-informed) machine learning reported in the recent literature, in which physical knowledge is incorporated into data-driven learning to improve prediction reliability under data-scarce conditions. Unlike conventional black-box machine learning algorithms, the proposed methodology embeds experimentally established deformation mechanisms, stage-wise parameterization, and physics-informed continuity constraints into the learning process while preserving mechanical interpretability [12,13,14]. The framework explicitly integrates physical deformation mechanisms, stage-wise segmentation, regression-based learning, and physics-informed constraints, thereby avoiding purely black-box modeling and ensuring mechanical consistency and interpretability. A schematic overview of the workflow is presented in Figure 1.

##### Experimental Dataset and Baseline Reference Curves

The ML framework was trained using experimentally obtained pull-out force–displacement data corresponding to two reference material states: nano-additive-free control fabrics (KPO, 0.0 wt.% nh-B_4_C) and nano-functionalized fabrics (BPO, 0.3 wt.% nh-B_4_C). For each specimen, the experimental pull-out response is expressed in Equation (7).

(7) F=F(x)

where *F* is the pull-out force (N) and *x* is the displacement (mm). These experimentally measured curves were treated as ground-truth reference responses for supervised learning. Pull-out responses for intermediate nano-additive contents (0.1–1.0 wt.%) were generated by generalizing the learned stage-wise regression parameters as continuous functions of the nh-B_4_C concentration. In the present study, prediction refers to the generation of physically admissible pull-out responses for untested nh-B_4_C concentrations based on experimentally validated reference states. Accordingly, the proposed PG-HML framework functions as a surrogate predictive model that enables the virtual exploration of nano-additive contents beyond the experimentally investigated compositions while preserving the underlying physical deformation mechanisms. The complete input dataset used by the proposed PG-HML framework is summarized in Table 2. The experimentally measured pull-out force–displacement curves corresponding to the KPO (0.0 wt.% nh-B_4_C) and BPO (0.3 wt.% nh-B_4_C) reference specimens under single-, two-, and three-yarn pull-out configurations constituted the only input data employed for supervised learning, parameter extraction, calibration, and validation. Subsequently, the experimentally calibrated stage-wise regression framework was used to predict pull-out responses for previously untested nh-B_4_C concentrations (0.1–1.0 wt.%), thereby enabling virtual exploration of nano-additive effects without requiring additional experimental measurements. In the context of the present study, the term “prediction” is used consistently with the supervised machine learning, surrogate modeling, and physics-guided machine learning literature, where models trained using experimentally observed input–output relationships are employed to estimate responses for previously unseen input conditions rather than merely reproducing the original training data. Accordingly, the proposed PG-HML framework predicts the pull-out responses of material compositions that were not experimentally characterized but remain within the physically consistent and mechanically admissible domain defined by the experimentally calibrated reference datasets.

Only the experimentally measured KPO (0.0 wt.%) and BPO (0.3 wt.%) pull-out force–displacement curves were used as input data for supervised learning, calibration, and validation. No experimental data corresponding to intermediate nh-B_4_C concentrations were used during model development. Instead, pull-out responses for intermediate concentrations (0.1–1.0 wt.%) were generated using the experimentally calibrated stage-wise PG-HML framework. Accordingly, these experimentally measured pull-out responses constitute the baseline dataset for supervised learning, parameter extraction, calibration, and validation of the proposed PG-HML framework, whereas the responses corresponding to intermediate nh-B_4_C concentrations are entirely model-generated.

##### Data Preprocessing and Signal Conditioning

Raw experimental force–displacement signals were subjected to preprocessing to improve numerical stability and preserve physically meaningful features. High-frequency noise was reduced using smoothing filters, such as moving-average filtering in Equation (8). The displacement axis was resampled onto a uniform grid in Equation (9). Normalization was applied only for comparative analysis and did not affect physical regression, as shown in Equations (10) and (11).

(8) $$\bar{F_{k}}=\frac{1}{2m+1}\;\sum_{i=-m}^{m}F_{k+i}$$

(9) $$x_{k}=x_{0}+k∆x$$

(10) $$x^{*}=\frac{x}{x_{max}}$$

(11) $$F^{*}=\frac{F}{F_{max}}$$

##### Physics-Based Stage-Wise Segmentation

Unlike conventional black-box machine learning approaches, the proposed physics-guided hybrid machine learning (PG-HML) framework combines supervised regression learning with physically meaningful deformation-stage decomposition and physics-informed continuity constraints. Rather than directly fitting the entire pull-out curve, the force–displacement response is separated into distinct mechanical stages, and each stage is modeled using an appropriate regression formulation. This strategy improves model interpretability, preserves physical consistency, and enables reliable prediction under limited experimental data conditions.

The stage-wise decomposition adopted in the present framework is based on experimentally established pull-out mechanisms widely reported for woven fabric structures, including crimp extension, initial interlacement rupture, and stick–slip sliding. These deformation mechanisms have been extensively documented in previous pull-out studies and constitute the physical foundation of the proposed PG-HML methodology. Accordingly, the implemented physics-informed continuity and admissibility constraints are directly derived from these accepted mechanical principles, ensuring physically realistic and mechanically consistent predictions. It should be noted that the term physics-guided in the present study does not imply the direct use of governing equations derived from first-principles frictional sliding or interfacial contact mechanics. Instead, physical knowledge is incorporated through experimentally established deformation mechanisms, mechanically meaningful state transitions, stage-specific parameterization, and continuity constraints that preserve physically admissible behavior throughout the pull-out process. Therefore, the proposed PG-HML framework should be regarded as a physically constrained surrogate modeling approach that bridges experimentally observed pull-out mechanisms with data-driven prediction under limited-data conditions. This implementation is consistent with the broader philosophy of physics-guided machine learning, whereby domain knowledge is embedded into the learning process to improve physical consistency, robustness, and predictive capability under limited training data [12,13,14].

Each pull-out force–displacement curve was decomposed into three physically meaningful stages. Stage I corresponds to crimp extension, characterized by yarn straightening and a gradual increase in pull-out force, as described by Equations (12) and (13). Stage II represents the initial interlacement rupture, during which the peak force and the first post-peak decay occur, as defined by Equation (14). Stage III denotes stick–slip sliding, governed by friction-controlled post-rupture yarn motion, as formulated in Equation (15).

(12) $$x\;\in \;\left[x_{0},\;x_{A}\right]$$

(13) $$F(x_{A})=F_{max}$$

(14) $$x\;\in \;\left[x_{A},\;x_{C}\right]$$

(15) $$x\;\in \;\left[x_{C},\;x_{end}\right]$$

where *x_A_* is the displacement at maximum pull-out force (peak point), and *x_C_* is the displacement at the first local minimum after the peak (first rupture minimum).

##### Stage-Wise Regression Modeling

Stage I—Crimp extension (cubic polynomial regression): The monotonic force increase during yarn straightening was modeled using a third-order polynomial, as shown in Equation (16). This formulation captures nonlinear crimp removal and progressive yarn straightening. The coefficients (*a*_0_, *a*_1_, *a*_2_, *a*_3_) were obtained via supervised least-squares regression.

(16) $$F_{I}(x)=a_{3}x^{3}+a_{2}x^{2}+a_{1}x+a_{0}$$

Stage II—Initial interlacement rupture (quadratic regression): The peak-force transition and interlacement rupture region were modeled using a second-order polynomial, as shown in Equation (17). This quadratic form captures the nonlinear force decay immediately after the peak pull-out force. Regression coefficients (*b*_0_, *b*_1_, *b*_2_) were obtained from supervised fitting. Physical continuity conditions were defined in Equations (18) and (19), ensuring smooth transitions between Stage I, Stage II and Stage III.

(17) $$F_{II}(x)=b_{2}x^{2}+b_{1}x+b_{0}$$

(18) $$F_{II}\left(x_{A}\right)=F_{max}$$

(19) $$F_{II}(x_{C})=F_{C}$$

Stage III—Stick–slip region (exponential decay model): The post-rupture friction-controlled sliding regime was modeled using a pure exponential decay function, as shown in Equation (20), where A is the initial amplitude of the stick–slip envelope and k is the decay coefficient governing frictional energy dissipation. This formulation represents the decreasing average frictional resistance during progressive yarn sliding. The parameters A and k were obtained via supervised regression. In addition, the stage continuity condition is defined in Equation (21).

(20) $$F_{III}=A\;exp(-kx)$$

(21) $$A\;exp(-kx_{C})=F_{II}(x_{C})$$

##### Physics-Informed Constraints

To ensure physical realism and mechanical consistency, the following constraints were imposed, as shown in Equations (22)–(24). These constraints enforce force continuity, suppress non-physical oscillations, and prevent unrealistic post-peak force drops.

(22) F(x)≥0

(23) $$F_{I}(x_{A})=F_{II}(x_{A})=F_{max}$$

(24) $$F_{II}(x_{C})=F_{III}(x_{C})$$

##### Concentration-Dependent Parameter Regression

Each stage-wise parameter vector is expressed in Equations (25) and (26) as a continuous function of nh-B_4_C concentration *c*, where *g*(*c*) was modeled using supervised regression. This enables the prediction of full pull-out curves for untested nh-B_4_C ratios without additional experiments.

(25) $$\theta =\left\{a_{i},b_{i},A,k\right\}$$

(26) θ(c)=g(c)

##### Model Validation and Output Generation

Model performance was evaluated using Equations (27)–(30) below.

(27) $$MSE=\frac{1}{n}\sum {\left(F_{pred}-F_{exp}\right)}^{2}$$

(28) $$MAE=\frac{1}{n}\sum \left|F_{pred}-F_{exp}\right|$$

(29) $$RMSE=\sqrt{\frac{1}{N}\sum_{i=1}^{N}{\left(y_{i}-{\hat{y}}_{i}\right)}^{2}}$$

(30) $$R^{2}=1-\frac{\sum {\left(F_{pred}-F_{exp}\right)}^{2}}{\sum {\left(F_{exp}-{\bar{F}}_{exp}\right)}^{2}}$$

where *MSE* is the mean square error, *MAE* is the mean absolute error, *RMSE* is the root of the mean square error, and *R*^2^ is the coefficient of regression. All computations, regression fitting, parameter extraction, curve generation, and figure production were performed in Python. The framework outputs include predicted pull-out curves, extracted mechanical parameters, energy metrics, and publication-ready figures. Model validation was performed by directly comparing the experimentally measured and PG-HML-predicted pull-out force–displacement curves for the KPO (0.0 wt.%) and BPO (0.3 wt.%) reference specimens under single-, two-, and three-yarn pull-out configurations. Validation included both quantitative evaluations using MSE, MAE, RMSE, and R^2^ metrics and qualitative assessment of the predicted curve morphology, including the crimp extension region, peak pull-out force, rupture transition, and stick–slip evolution. The validated stage-wise regression framework was subsequently used to generate physically admissible pull-out responses for previously untested nh-B_4_C concentrations.

## 3. Results and Discussion

### 3.1. Experimental and Machine Learning-Based Characterization of Nano-Coated Fabric Pull-Out Behavior

**Figure 2:**
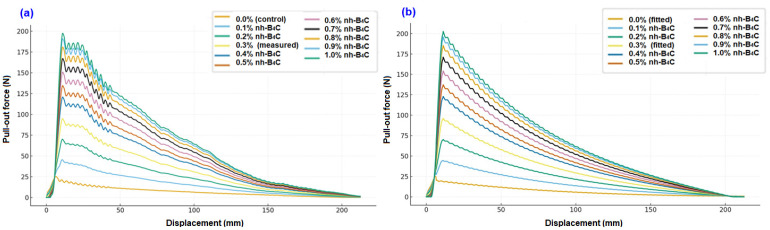
Single-yarn pull-out force–displacement behavior of nano-coated p-aramid substrate as a function of nh-B_4_C content, obtained using a machine learning-assisted modeling framework. (**a**) Experimentally measured KPO (0.0 wt.% nh-B_4_C) and BPO (0.3 wt.% nh-B_4_C) pull-out responses, together with the corresponding PG-HML predictions used for model validation; and (**b**) PG-HML predictions for previously untested nh-B_4_C concentrations (0.1–1.0 wt.%).

**Figure 3:**
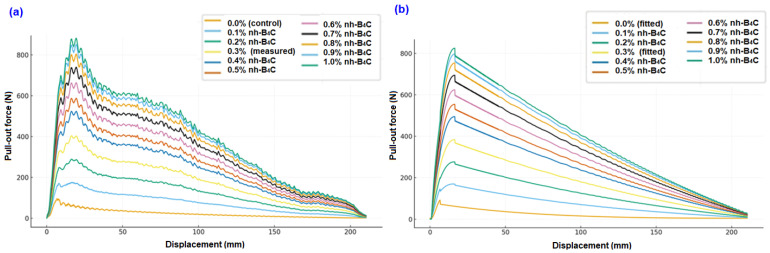
Two-yarn pull-out force–displacement behavior of para-aramid fabric systems as a function of nh-B_4_C content, obtained using a machine learning-assisted modeling framework. (**a**) Experimentally measured KPO (0.0 wt.% nh-B_4_C) and BPO (0.3 wt.% nh-B_4_C) pull-out responses, together with the corresponding PG-HML predictions used for model validation; and (**b**) PG-HML predictions for previously untested nh-B_4_C concentrations (0.1–1.0 wt.%).

**Figure 4:**
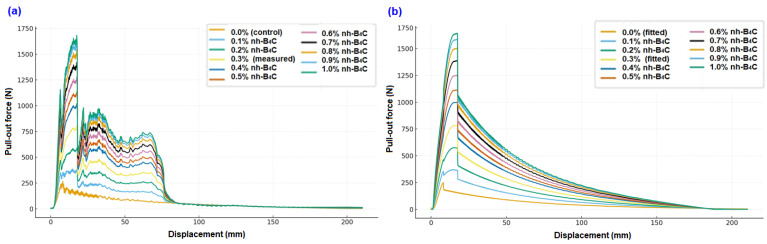
Three-yarn pull-out force–displacement behavior of para-aramid fabric systems as a function of nh-B_4_C content, obtained using a machine learning-assisted modeling framework. (**a**) Experimentally measured KPO (0.0 wt.% nh-B_4_C) and BPO (0.3 wt.% nh-B_4_C) pull-out responses, together with the corresponding PG-HML predictions used for model validation; and (**b**) PG-HML predictions for previously untested nh-B_4_C concentrations (0.1–1.0 wt.%).

The pull-out response of both control (KPO) and nh-B_4_C-coated (BPO) para-aramid fabrics exhibits a consistent three-stage behavior: crimp extension (Stage I), initial interlacement rupture (Stage II), and stick–slip sliding (Stage III) [85,86]. Stage I reflects progressive yarn straightening and stiffness evolution, Stage II corresponds to peak force attainment and rupture transition, and Stage III is governed by friction-controlled oscillatory sliding. A physics-guided hybrid supervised machine learning framework was developed using experimental KPO (0.0 wt.%) and BPO (0.3 wt.%) datasets as anchor references to predict pull-out behavior across 0.1–1.0 wt.% nh-B_4_C contents. Stage-wise regression was implemented to preserve mechanical interpretability, employing polynomial functions for Stages I–II and an exponential decay formulation for the post-peak stick–slip regime. Model accuracy was evaluated using MSE, MAE, RMSE, and R^2^ metrics, demonstrating strong agreement with experimental data, particularly in the mechanically critical pre-peak and rupture regions. Accordingly, the agreement between experimentally measured and PG-HML-predicted responses shown in Figure 2a, Figure 3a and Figure 4a constitutes the primary validation of the proposed framework, whereas the remaining predicted responses were generated using the experimentally calibrated stage-wise regression model. Moreover, the experimentally measured KPO and BPO pull-out curves shown in Figure 2a, Figure 3a and Figure 4a serve as the primary reference responses for validating the proposed PG-HML framework before the prediction of previously untested nh-B_4_C concentrations. Further, the experimentally measured KPO (0.0 wt.%) and BPO (0.3 wt.%) pull-out responses presented in Figure 2a, Figure 3a and Figure 4a constitute the reference datasets used for the validation of the proposed PG-HML framework, whereas the remaining curves represent model predictions for previously untested nano-additive concentrations.

#### 3.1.1. Experimental and Machine Learning-Predicted Single- and Multi-Yarn Pull-Out Force–Displacement

Table 3 and Figure 2 present the machine learning-predicted single-yarn pull-out force–displacement parameters as a function of nh-B_4_C content.

As shown in Figure 2a,b, nano-coated fabrics exhibit significantly higher pull-out forces than the control across all stages, particularly in the crimp extension and rupture regions. The ML framework reproduces the control and 0.3 wt.% experimental responses with high accuracy (≤1% deviation in peak force), confirming reliable capture of stiffness evolution and peak formation. Maximum pull-out force increases monotonically from ~25 N (0.0 wt.%) to ~198 N (1.0 wt.%), whereas peak displacement rises initially and then stabilizes (~10.9–11.0 mm). This indicates that nano-reinforcement primarily enhances frictional load transfer and interfacial resistance rather than deformation capacity. Stage I stiffness increases systematically with nh-B_4_C content, reflecting enhanced surface roughness and filament–particle interlocking. The Stage II rupture force rises markedly (19.2 N to 177.2 N), demonstrating that interlacement failure becomes the dominant load-bearing mechanism. The accurate prediction of both F_max_ and rupture force (F_C_) confirms that physically meaningful scaling is embedded in the ML formulation. In Stage III, increasing nh-B_4_C shifts the stick–slip response to a higher force envelope, while the model prioritizes continuity and physically admissible post-peak decay over local oscillations. Overall, the physics-guided framework preserves key mechanical descriptors—maximum force, rupture force, and intra-yarn shear strength—ensuring accurate and mechanically consistent predictions across the investigated nh-B_4_C range.

Table 3 and Figure 3 and Figure 4 present the machine learning-predicted multi-yarn (two- and three-yarn) pull-out force–displacement responses as a function of nh-B_4_C content. Compared to single-yarn extraction, multi-yarn configurations exhibit substantially higher force levels and more pronounced nonlinear behavior, reflecting the collective load-sharing and frictional interactions that are critical for soft ballistic fabric performance [88].

For two-yarn pull-out (Table 3 and Figure 3a,b), the model reproduces the control response with high accuracy (2–3% deviation in peak force and 7% in displacement). For the 0.3 wt.% composition, peak force is predicted within 5%, although peak displacement is moderately underestimated, indicating that the framework prioritizes force scaling over localized deformation near the maximum. Increasing the nh-B_4_C content systematically enhances Stage I stiffness and Stage II rupture force, reflecting strengthened inter-yarn interactions. Stage segmentation based on the force peak (x_A_) and first post-peak minimum (x_C_) ensures that mechanical transition points are physically encoded. In Stage III, higher nh-B_4_C contents shift the stick–slip response to elevated force envelopes, while the fitted curves preserve smooth, physically admissible decay rather than replicating stochastic oscillations.

For three-yarn pull-out (Table 3 and Figure 4a,b), predictive performance remains excellent. For the control and 0.3 wt.% configurations, both peak force and displacement are reproduced with <1% errors, confirming accurate capture of force magnitude and deformation morphology in a highly coupled system. Across the investigated nh-B_4_C range, Stage I stiffness and Stage II rupture forces increase monotonically, whereas maximum displacement stabilizes at higher contents. Defining stage boundaries at F_max_ (x_A_) and the first major post-peak minimum (F_C_ at x_C_) maintains physically meaningful segmentation and prevents numerical artefacts. Overall, the ML framework preserves consistent force scaling, stage continuity, and monotonic parameter evolution, demonstrating global mechanical coherence in multi-yarn pull-out prediction. It should be noted that the agreement between experimental and predicted responses decreases slightly within the later portion of Stage III, particularly for two- and three-yarn pull-out configurations. This behavior is attributed to stochastic stick–slip interactions associated with repeated yarn re-engagement, local interlacement release, filament rearrangement, and fluctuating frictional contact conditions. Since the present PG-HML framework represents Stage III using a physics-constrained exponential decay envelope, the model captures the global trend of frictional force decay and energy dissipation while smoothing local high-frequency oscillations. Consequently, minor deviations may occur at larger pull-out displacements, although the principal mechanical descriptors and overall response evolution remain accurately represented.

**Figure 5:**
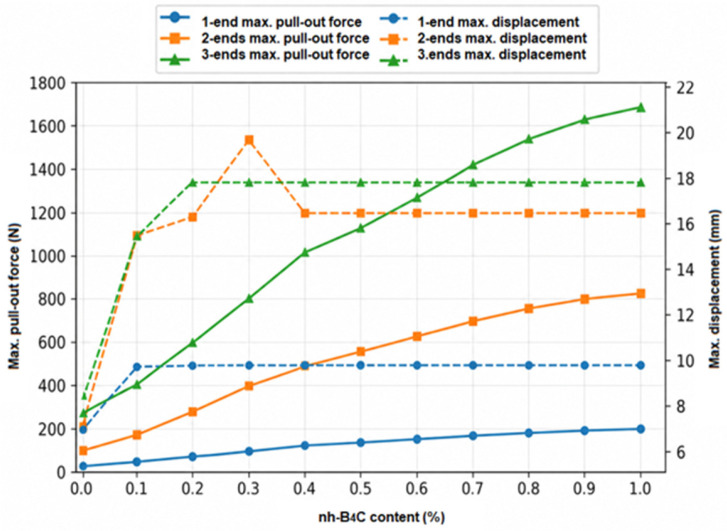
Machine learning-assisted predictions of maximum pull-out force–displacement for single- and multiple-yarn configurations as a function of nh-B_4_C content.

**Figure 6:**
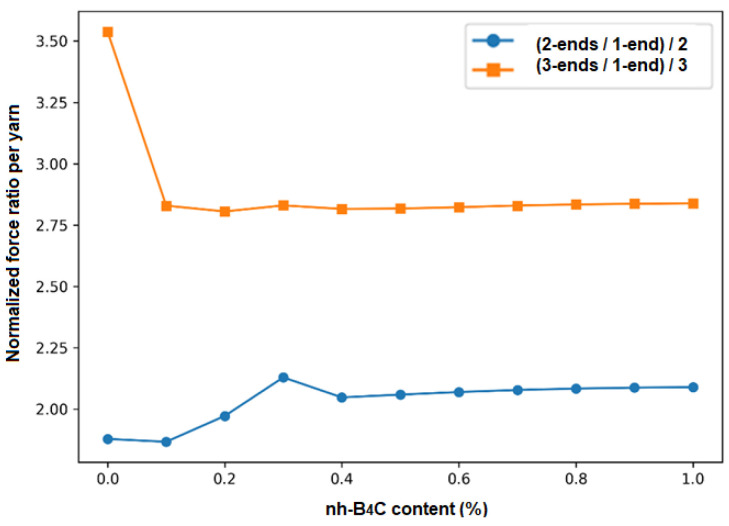
Machine learning-assisted predictions of normalized force ratio per yarn for pull-out yarn end configurations as a function of nh-B_4_C content.

Figure 5 and Figure 6 summarize the machine learning-assisted predictions of maximum pull-out force–displacement and normalized force ratio per yarn for single- and multiple-yarn configurations as a function of nh-B_4_C content.

For all configurations, increasing nh-B_4_C systematically enhances the maximum pull-out force, whereas the displacement response depends on the yarn number. In the single-yarn configuration, the maximum force increases from ~30–35 N (0.0 wt.%) to ~200 N (1.0 wt.%), corresponding to a ~500–550% enhancement, while displacement rises more moderately (~6.5 mm to ~11 mm). This indicates that nano-reinforcement primarily strengthens interfacial friction and Stage I stiffness without substantially extending deformation capacity. For two-yarn pull-out, the effect is more pronounced: maximum force increases from ~100 N to ~800–850 N (~700–750%), whereas displacement increases rapidly at low contents and then plateaus (~16–17 mm). This saturation suggests that, beyond the Stage II transition, further force gains are governed by frictional strengthening rather than proportional deformation growth. The three-yarn configuration shows the highest absolute force levels, increasing from ~270 N to ~1650–1700 N (~500–530%), reflecting strong synergy between nano-reinforcement and multi-yarn load sharing. Despite this substantial force amplification, maximum displacement stabilizes (~17–19 mm), confirming that post-peak behavior remains dominated by friction-controlled sliding rather than continued extension.

Figure 6 illustrates the effect of nh-B_4_C content on load-sharing efficiency through normalized force ratios per yarn. In the two-yarn system, the normalized ratio increases from ~1.85–1.90 (0.0 wt.%) to ~2.1 (~0.3 wt.%) and then stabilizes (~2.05–2.10), corresponding to a ~10–15% increase in load carried per yarn relative to single-yarn pull-out. For the three-yarn configuration, the normalized ratio decreases from ~3.5 (0.0 wt.%) to ~2.8–2.9 beyond 0.1 wt.% and remains nearly constant thereafter, indicating a transition toward more uniform load sharing with increasing nano-reinforcement. The observed plateau behavior confirms saturation of inter-yarn friction and mechanical interlocking at higher nh-B_4_C contents. Importantly, this stabilization emerges naturally from the data-driven ML framework, demonstrating accurate learning of force scaling and load sharing evolution across yarn configurations.

#### 3.1.2. Machine Learning-Predicted Regression of Single- and Multi-Yarn Pull-Out

**Figure 7:**
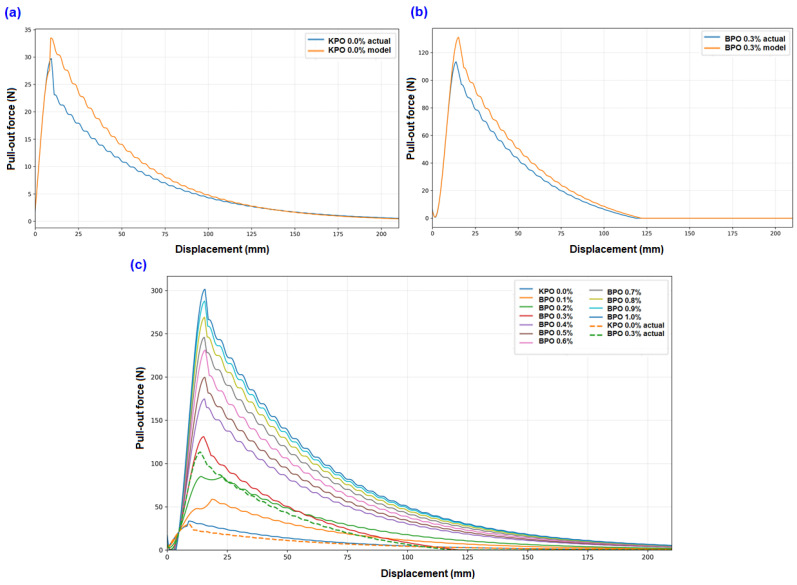
Stage-integrated single-yarn pull-out force–displacement curves reconstructed using the experimentally validated stage-wise physics-guided hybrid machine learning (PG-HML) framework for para-aramid fabrics containing different nh-B_4_C concentrations. The complete responses were obtained by integrating the individually modeled crimp extension, initial interlacement rupture, and stick–slip stages after calibration and validation against experimentally measured KPO (0.0 wt.%) and BPO (0.3 wt.%) pull-out data. (**a**) Control (KPO), (**b**) nh-B_4_C-coated (BPO), and (**c**) concentration-dependent variation in the predicted pull-out behavior.

**Figure 8:**
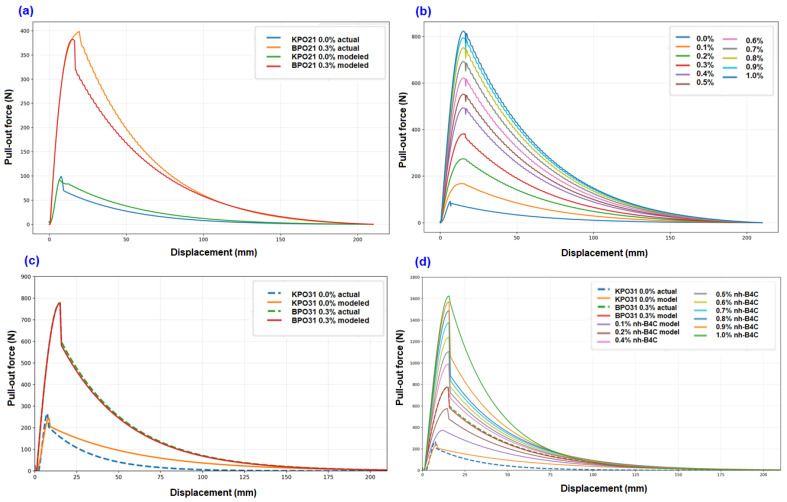
Integrated-stage force–displacement curves for multiple-yarn pull-out behavior constructed using experimental data and machine learning-based models for p-aramid fabrics. (**a**) Two-yarn control (KPO) and nh-B_4_C-modified specimens (BPO), (**b**) two-yarn variation with nh-B_4_C content, (**c**) three-yarn control (KPO) and nh-B_4_C-modified specimens (BPO), and (**d**) three-yarn variation across different nh-B_4_C levels.

Table 4 summarizes the stage-dependent machine learning-assisted regression models describing the single- and multi-yarn pull-out force–displacement response of nh-B_4_C-functionalized p-aramid fabrics, while Table 5 presents their quantitative performance metrics. Figure 7a–c illustrate the stage-integrated force–displacement curves reconstructed from experimental data and ML-based regression models. Figure 8a–d illustrate the corresponding stage-integrated force–displacement curves reconstructed from experimental data and ML-based regression equations.

In single-yarn pull-out, Stage I (crimp extension) is accurately described by third-order polynomials (R^2^ ≈ 0.988–0.997). With increasing nh-B_4_C content, the cubic coefficient becomes more negative and the linear terms increase, indicating sharper peak transitions and elevated force levels due to enhanced inter-filament friction and surface roughness. Stage II (interlacement rupture) follows a quadratic regression (R^2^ ≈ 0.982–0.994). The quadratic coefficient shifts toward stronger negative curvature with increasing nh-B_4_C, reflecting intensified load transfer and more abrupt rupture transitions. Despite the mechanical sensitivity of this stage, prediction accuracy remains consistently high. Stage III (stick–slip) is represented by an exponential decay, F(x) = A exp(−kx). The decay constant remains nearly invariant (k ≈ 0.020), indicating that post-peak decay length is governed primarily by pull-out kinematics. In contrast, the amplitude A increases markedly (28.4 to 376.2), demonstrating strong enhancement of frictional force levels with nh-B_4_C addition. Although this regime is inherently stochastic, R^2^ values (~0.77–0.83) confirm reliable envelope prediction. In two-yarn pull-out, Stage I regression remains highly accurate (R^2^ ≈ 0.998–0.999), confirming the highly reproducible and physically consistent behavior of the initial crimp extension stage.

Stage II performance improves with increasing nh-B_4_C, reaching near-unity R^2^ at ≥0.4 wt.%, indicating stabilization of rupture mechanisms under nano-reinforcement. Stage III decay constants converge (~0.013–0.017), suggesting a common friction-controlled functional form across compositions, with R^2^ ≈ 0.85–0.88 capturing the global envelope despite stick–slip variability. In three-yarn pull-out, Stage I maintains high regression accuracy, though coefficient sensitivity increases due to greater contact density and load-sharing complexity. Stage II shows higher variability at low nh-B_4_C contents but approaches R^2^ ≳ 0.98–0.999 at elevated nh-B_4_C concentrations, indicating a progressively more stable and physically consistent rupture response. Stage III exponential models preserve consistent post-peak envelopes; increasing amplitude reflects elevated force levels, while moderate R^2^ values (~0.71–0.93) capture engineering-relevant trends without overfitting micro-scale oscillations.

The error metrics In Table 5 further confirm this behavior. In Stages I and II, low MSE, MAE, and RMSE values accompany the high R^2^, demonstrating precise prediction of early deformation and rupture-controlled regimes. In stage III, absolute error values increase with nh-B_4_C content due to the substantial rise in force magnitude; however, the corresponding R^2^ values remain relatively stable, indicating that the model continues to explain a large fraction of the response variance. Thus, the increase in error primarily reflects scale effects rather than loss of predictive capability. Error metrics in Table 5 further support these observations. In both two- and three-yarn systems, substantial reductions in MSE, MAE, and RMSE are achieved in Stages I and II when transitioning from experimental baselines to ML-based models, accompanied by corresponding increases in R^2^. In Stage III, absolute errors increase with force magnitude; however, R^2^ values remain stable, indicating preserved explanatory power despite scale effects. Notably, in several cases (e.g., BPO 0.3 wt.%), the ML model yields higher stage III R^2^ than the experimental baseline, demonstrating effective suppression of noise and improved extraction of the underlying trend.

Figure 7 corroborates these findings at the curve level. For the control fabric, discrepancies between experimental and predicted curves are mainly confined to Stage III, consistent with the more irregular stick–slip behavior of the unmodified fabric. In contrast, the nh-B_4_C-coated fabric (0.3 wt.%) exhibits markedly improved agreement across all stages, with near-perfect overlap in Stages I and II and substantially improved consistency in Stage III. When all compositions are plotted together (Figure 7c), increasing nh-B_4_C content results in a systematic upward shift of the force envelope, particularly in the post-peak regime, in direct agreement with the increase in the exponential amplitude A reported in Table 4.

As a whole, the stage-dependent ML-assisted regression framework successfully converts limited experimental data into closed-form force–displacement relationships. The high accuracy achieved in Stages I and II enables reliable identification of stiffness evolution and rupture thresholds, while the robust trend prediction in Stage III supports friction- and energy-based optimization. Consequently, these regression models provide a practical design tool, allowing rapid estimation of full pull-out curves, peak force, and energy absorption for arbitrary nh-B_4_C contents without additional experiments, thereby significantly reducing experimental workload and accelerating material optimization. From an engineering perspective, the experimentally validated PG-HML framework enables virtual generation of complete pull-out force–displacement responses and associated mechanical descriptors for previously untested nh-B_4_C concentrations. Therefore, the influence of nano-additive content on pull-out performance can be systematically explored before conducting additional experimental investigations, thereby substantially reducing the experimental effort required during preliminary material screening and optimization.

Figure 8a–d visually exhibit the quantitative analysis. Experimental and ML-predicted curves show strong overlap in Stages I and II for both KPO and BPO specimens, while post-peak deviations are largely confined to the stick–slip region. The curve families in Figure 8b,d clearly demonstrate the systematic evolution of peak force, initial stiffness, and post-peak decay with increasing nh-B_4_C content. Crucially, the closed-form regression equations in Table 4 enable rapid generation of complete force–displacement curves and direct calculation of performance metrics (e.g., peak force and energy absorption) across the entire composition range. Overall, the stage-dependent ML-assisted regression framework effectively transforms limited experimental multi-yarn pull-out data into a scalable and physically consistent predictive tool. High accuracy in Stages I and II ensures reliable characterization of stiffness evolution and rupture thresholds, while robust trend capture in Stage III supports friction- and energy-based design optimization, even in mechanically complex multi-yarn configurations.

#### 3.1.3. Machine Learning-Assisted Prediction of Initial Interlacement Rupture Forces in Nano-Coated Fabric Materials

**Figure 9:**
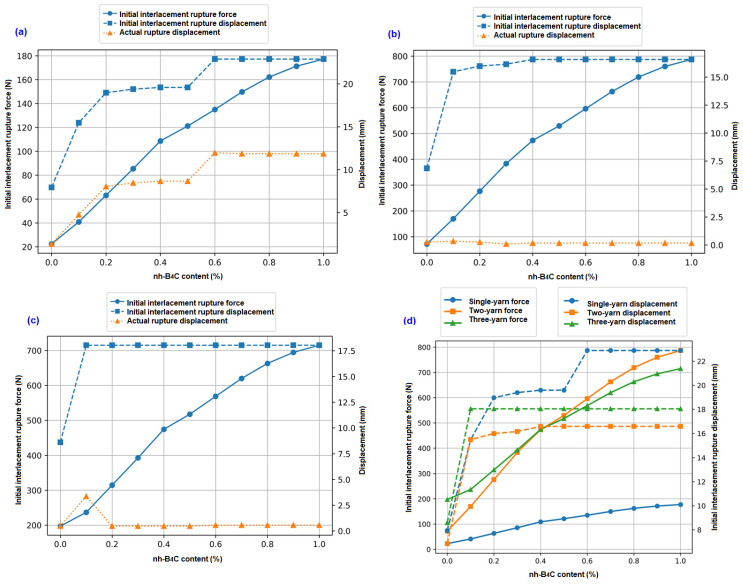
Machine learning-assisted force–displacement characteristics corresponding to the initial interlacement rupture stage in single- and multi-yarn pull-out behavior of nh-B_4_C-modified p-aramid material. (**a**) Single-yarn, (**b**) two-yarn, (**c**) three-yarn, and (**d**) combined comparison of all yarn configurations.

Figure 9a–d present the machine learning-assisted predictions of force–displacement characteristics corresponding to the initial interlacement rupture stage for single-, two-, and three-yarn pull-out configurations of nh-B_4_C-modified p-aramid fabrics. For the single-yarn system (Figure 9a), rupture force increases monotonically from 19.20 N (0.0 wt.%) to 86.47 N at 0.3 wt.% (experimental), with the ML prediction (85.42 N) deviating by <2%. The model forecasts continued growth to 177.22 N at 1.0 wt.%. In contrast, rupture displacement increases moderately and then plateaus (~22–23 mm), indicating that nano-reinforcement primarily enhances interfacial strength rather than delaying rupture onset. In the two-yarn configuration (Figure 9b), rupture force rises from 63.37 N to 377.05 N at 0.3 wt.% (an approximately six-fold increase), with prediction error below 2%. At 1.0 wt.%, the predicted rupture force reaches 786.83 N, reflecting the combined effect of nanoparticle-induced friction and multi-yarn confinement. Rupture displacement stabilizes (~15–16 mm), suggesting a transition to a force-dominated rupture regime consistently captured by the model. For three-yarn pull-out (Figure 9c), rupture forces are highest due to increased confinement, increasing from 180.19 N to 329.24 N at 0.3 wt.%, and reaching 714.99 N at 1.0 wt.% in prediction. Although minor deviations occur at intermediate contents, overall trend fidelity remains strong. Rupture displacement remains nearly constant (~18 mm), indicating force-controlled instability rather than progressive geometric rearrangement. The combined comparison (Figure 9d) confirms that rupture force scales with yarn number (single < two < three) for all nh-B_4_C contents, reflecting cumulative frictional interaction and load sharing. The ML framework preserves this hierarchy and its monotonic evolution, providing physically consistent rupture predictions across configurations while substantially reducing experimental requirements.

The predicted stage-wise mechanical descriptors presented in Figure 9 demonstrate that the proposed PG-HML framework provides more than complete force–displacement curves. The framework also enables quantitative estimation of physically meaningful parameters governing interfacial load transfer and energy dissipation, including pull-out force, rupture characteristics, pull-out energy, and intra-yarn shear strength. These quantities can therefore be employed for preliminary evaluation and optimization of nano-functionalized protective textile materials prior to additional experimental testing.

#### 3.1.4. Machine Learning Modeling of Single- and Multi-Yarn Stick–Slip Behavior in Nano-Coated Fabrics

**Figure 10:**
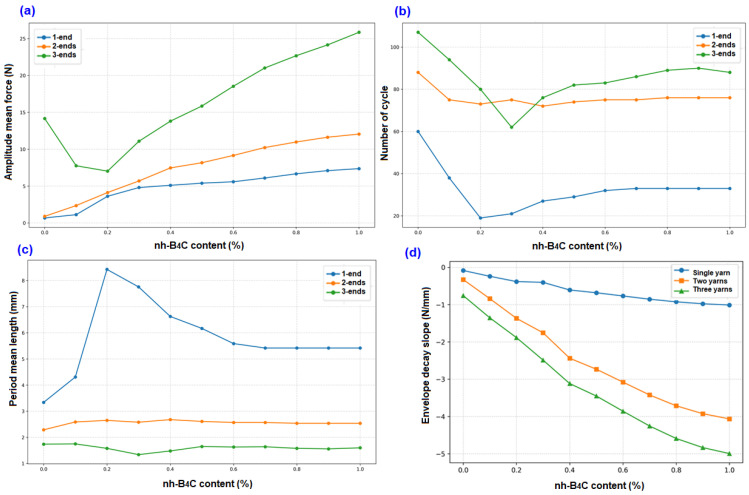
Stick–slip response metrics predicted using machine learning-assisted models for aramid fabrics with varying nh-B_4_C ratios. (**a**) Mean force amplitude, (**b**) number of stick–slip cycles, (**c**) mean period length, and (**d**) envelope decay slope for single- and multi-yarn pull-out behavior.

Stick–slip represents the most stochastic and friction-dominated stage of the pull-out response, governed by intermittent interfacial locking and release events. Table 6 presents the ML-assisted characterization of stick–slip parameters for single-, two-, and three-yarn configurations, while Figure 10a–c exhibit the corresponding trends as a function of nh-B_4_C content.

For the single-yarn configuration (Table 6 and Figure 10a–d), the stick–slip regime extends over a wide displacement range (>9–11 mm to >210 mm), indicating prolonged frictional sliding. Increasing nh-B_4_C content reduces the number of cycles (~97 to ~20–33), marking a shift from frequent low-amplitude oscillations to fewer, higher-energy slip events. Correspondingly, mean and maximum force amplitudes increase, reaching ~7–8 N and ~47 N at 1.0 wt.%. Despite this amplification, the dominant frequency and wavelength remain nearly constant (~0.005 cycles/mm and ~200 mm), indicating that nano-reinforcement intensifies slip magnitude without altering the intrinsic oscillation scale. In the two-yarn configuration (Table 6, Figure 10a–d), stick–slip behavior becomes more energetic due to increased contact multiplicity. Cycle numbers remain relatively high (~70–115), while force amplitudes rise sharply (ΔF from ~3 N to >12 N; ΔF_max_ > 340 N at 1.0 wt.%). Mean period length remains stable (~2.5–2.7 mm), whereas maximum period length expands (~80 mm), indicating intermittent large slip events superimposed on a stable local spacing. Increasingly negative envelope slopes (down to ~−4 N/mm) confirm enhanced post-peak energy dissipation. For three-yarn pull-out, sliding is the most constrained and dissipative. Cycle numbers remain high (~80–107), while ΔF increases to ~26 N at 1.0 wt.% after minor non-monotonic behavior at intermediate contents. Shorter mean period lengths (~1.3–1.7 mm) reflect frequent local slip events under dense inter-yarn interactions. The steepest envelope slopes (≈−5 N/mm) indicate superior frictional energy dissipation. As in other configurations, frequency and wavelength remain nearly invariant, confirming that nh-B_4_C modifies slip intensity and decay rather than fundamental oscillatory spacing.

Figure 10a–d consolidate these trends, showing that mean force amplitude increases systematically with nh-B_4_C content across all yarn configurations, following the hierarchy single < two < three yarns. The evolution of cycle number and period length further demonstrates that nano-functionalization shifts the stick–slip response from a high-cycle, low-amplitude regime to a lower-cycle, high-amplitude, strongly dissipative regime, with the effect becoming more pronounced as yarn multiplicity increases. In general, Table 6 and Figure 10a–d demonstrate that the ML-assisted framework provides a consistent and quantitative description of stick–slip behavior across different pull-out configurations. By explicitly predicting force amplitudes, cycle density, and decay characteristics, the model enables a priori evaluation of frictional stability and energy dissipation without exhaustive experimentation. This capability allows rapid optimization of nh-B_4_C content, significantly reducing experimental cost and time while preserving strong physical interpretability, an outcome of direct relevance for materials design and performance-driven optimization.

Collectively, Figure 2, Figure 3, Figure 4, Figure 5, Figure 6, Figure 7, Figure 8 and Figure 9 show that the proposed PG-HML framework provides physically interpretable outputs beyond curve reconstruction. The predicted maximum pull-out force, rupture characteristics, intra-yarn shear strength, displacement response, and pull-out energy directly reflect interfacial load transfer and energy dissipation mechanisms in the nano-functionalized para-aramid fabrics. Thus, the practical role of the framework is visible through its ability to estimate key pull-out descriptors for previously untested nh-B_4_C concentrations, enabling preliminary virtual material screening while reducing the need for exhaustive experimental testing.

### 3.2. Machine Learning-Predicted Intra-Yarn Shear Strength as a Function of Nano-Content and Yarn Interaction

**Figure 11:**
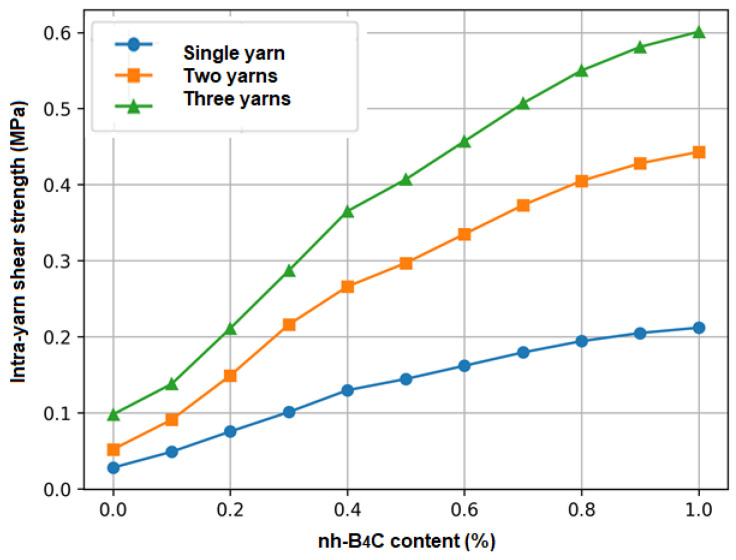
Intra-yarn shear strength responses of single- and multi-yarn pull-out configurations in nh-B_4_C-modified aramid fabrics predicted using machine learning-assisted models.

Figure 11 shows the machine learning-predicted evolution of intra-yarn shear strength as a function of nh-B_4_C content for single-, two-, and three-yarn pull-out configurations. The results extracted from Table 3 consistently demonstrate that intra-yarn shear strength is governed by the combined effects of nanoparticle functionalization and yarn interaction density.

For the single-yarn configuration, intra-yarn shear strength increases monotonically with nh-B_4_C content, rising from ~0.028 MPa (0.0 wt.%) to ~0.10 MPa at 0.3 wt.% and ~0.21 MPa at 1.0 wt.%. This trend reflects enhanced filament–filament friction and nano-bridging effects, with gradual saturation at higher contents. The smooth evolution confirms that shear strength is a stable and readily learnable parameter within the ML framework. In the two-yarn configuration, shear strength is consistently higher due to additional inter-yarn constraints. Values increase from ~0.05 MPa to ~0.22 MPa (0.3 wt.%) and ~0.44 MPa (1.0 wt.%), representing more than an eight-fold enhancement over the uncoated state. The steeper scaling compared to single-yarn pull-out indicates a synergistic amplification of nanoparticle-induced friction under multi-yarn interaction. The three-yarn system exhibits the highest shear strength, increasing from ~0.10 MPa to ~0.29 MPa (0.3 wt.%) and ~0.60 MPa (1.0 wt.%). A consistent hierarchy (single < two < three) is maintained across all contents, with increasing separation at higher nano loadings. This behavior confirms that nanoparticle functionalization and yarn multiplicity act synergistically to enhance collective load transfer and suppress filament sliding. Generally, Figure 11 demonstrates that the ML-assisted framework reliably captures both monotonic nano-content dependence and nonlinear amplification due to yarn interactions, providing a rapid and physically consistent tool for predicting shear strength across configurations.

### 3.3. Machine Learning-Driven Prediction of Pull-Out Energy Components in Nano-Coated Fabrics

**Figure 12:**
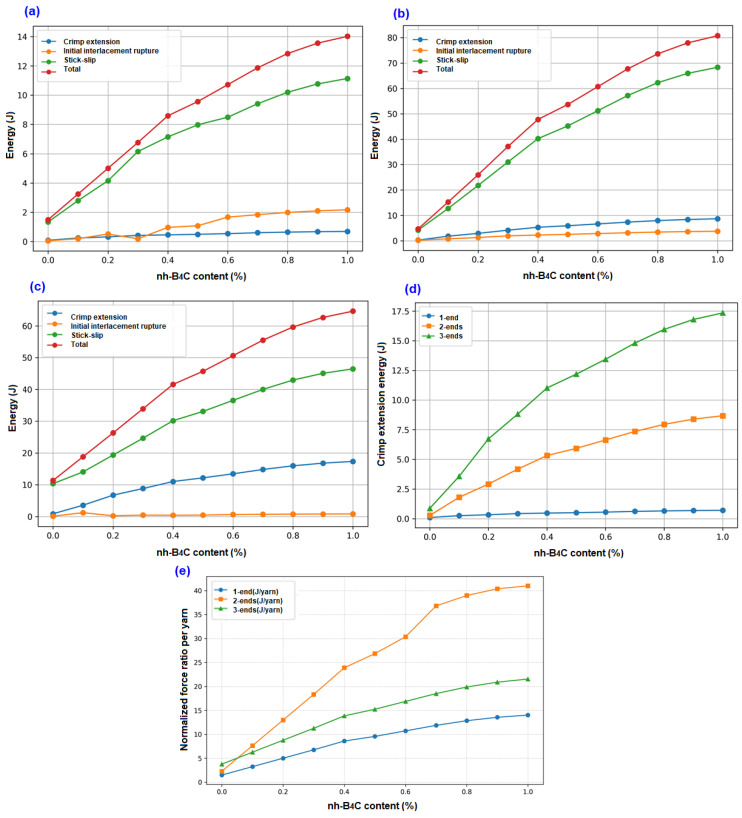
Pull-out energy components predicted using machine learning-assisted models for p-aramid fabrics with varying nh-B_4_C contents. (**a**) Total pull-out energy for single-, (**b**) two-, and (**c**) three-yarn configurations, (**d**) crimp extension energy contribution, and (**e**) normalized energy response across all yarn configurations.

Table 7 presents the experimentally measured and machine learning-predicted pull-out energy components for single- and multi-yarn p-aramid fabrics as a function of nh-B_4_C content, while Figure 12a–e present the corresponding trends and normalized comparisons. Across all configurations, the ML framework accurately reproduces experimental energy values and enables continuous prediction over the full nano-content range.

For the single-yarn configuration (Table 7 and Figure 12a), total pull-out energy increases from 1.50 J (0.0 wt.%) to 6.77 J at 0.3 wt.% and ~14.0 J at 1.0 wt.%, with ML predictions closely matching experimental values. Energy partitioning shows that this increase is primarily governed by stick–slip dissipation, while crimp extension and rupture energies remain comparatively minor. Thus, nano-functionalization mainly enhances friction-controlled sliding in single-yarn pull-out. In the two-yarn configuration (Table 7 and Figure 12b), total energy rises from 4.70 J to ~37.1 J (0.3 wt.%) and ~80.8 J (1.0 wt.%), again with strong prediction accuracy. Although stick–slip energy remains dominant, crimp extension contributes more significantly than in the single-yarn case, reflecting enhanced resistance to yarn straightening under inter-yarn constraint. The three-yarn system exhibits the highest energy absorption (Table 7 and Figure 12c), increasing from 11.3 J to ~33.9 J (0.3 wt.%) and ~64.6 J (1.0 wt.%). In this highly constrained configuration, crimp extension accounts for a substantial portion of total energy, particularly at higher contents; however, stick–slip dissipation remains the principal mechanism. Figure 12d confirms a hierarchy in crimp extension energy (three > two > single), which becomes more pronounced with increasing nh-B_4_C content. Figure 12e shows that normalized energy per yarn increases sub-linearly with yarn number, indicating diminishing marginal energy efficiency due to load sharing. Mostly, nh-B_4_C functionalization systematically enhances pull-out energy across configurations, and the ML framework reliably captures both total energy and its stage-wise distribution.

### 3.4. Comparative Evaluation of Machine Learning Models

#### 3.4.1. Single-Yarn Pull-Out Force–Displacement Modeling and Prediction

**Figure 13:**
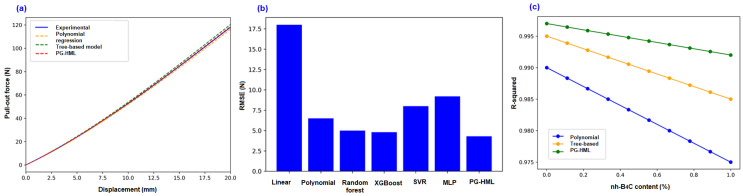
Comparative performance of machine learning models for single-yarn pull-out force–displacement prediction. (**a**) Experimental versus predicted responses, (**b**) RMSE-based model comparison, and (**c**) variation of prediction accuracy (R^2^) with nh-B_4_C content.

Figure 13a–c present a comparative evaluation of different machine learning approaches for predicting the single-yarn pull-out force–displacement response of nh-B_4_C-functionalized para-aramid fabrics. As shown in Figure 13a, all models reproduce the overall nonlinear trend of the experimental response; however, clear differences arise in their ability to capture mechanically critical features, particularly near the peak force and throughout the post-peak regime. Polynomial regression provides an acceptable approximation of the global force–displacement relationship but exhibits increasing deviation at higher displacements, where friction-dominated mechanisms become more pronounced. Tree-based models improve local fitting accuracy around the peak force; however, their predictions show reduced smoothness and stability in the post-rupture region, occasionally leading to non-physical fluctuations. In contrast, the physics-guided hybrid machine learning (PG-HML) framework demonstrates the closest agreement with experimental data across the entire displacement range, preserving both the pre-peak stiffness evolution and the physically consistent post-peak decay envelope. The quantitative comparison shown in Figure 13b supports these observations. Conventional linear and polynomial models yield relatively higher prediction errors, whereas tree-based, kernel-based, and neural network-based models achieve improved accuracy but remain sensitive to local data variability. Among all evaluated approaches, the PG-HML framework consistently exhibits the lowest RMSE under identical preprocessing and validation conditions, indicating superior predictive fidelity. Figure 13c illustrates the variation of the coefficient of determination (R^2^) with nh-B_4_C content. A gradual decrease in prediction accuracy is observed for all models as nano-additive content increases, reflecting the enhanced nonlinearity and frictional complexity introduced by nh-B_4_C functionalization. Despite this trend, the PG-HML model maintains a high and stable R^2^ across the full concentration range, highlighting its robustness against increasing interfacial interaction strength. Overall, this comparative analysis indicates that purely data-driven models are effective in capturing general force–displacement trends but may struggle to ensure mechanical consistency in the rupture and stick–slip regimes. By explicitly incorporating stage-wise physical constraints, the PG-HML framework provides a more reliable and physically admissible representation of single-yarn pull-out behavior, making it particularly suitable for data-efficient modeling and parametric exploration of nano-functionalized fiber interfaces in aramid fabric structures.

#### 3.4.2. Multi-Yarn Pull-Out Force–Displacement Modeling and Prediction

The predictive performance of the machine learning models was further evaluated for multi-yarn pull-out configurations, including two-yarn and three-yarn pull-out, where collective frictional interactions and load-sharing mechanisms dominate the mechanical response. Compared to single-yarn extraction, multi-yarn pull-out introduces increased nonlinearity, stronger inter-yarn coupling, and higher force levels, thereby providing a more stringent test for model robustness and physical consistency. Table 8 summarizes the quantitative performance metrics of the evaluated models for multi-yarn pull-out prediction. For both two-yarn and three-yarn configurations, polynomial regression captures the general force–displacement trend but exhibits increased error levels, particularly in the post-peak regime, where cumulative frictional effects and stick–slip interactions intensify. Tree-based and neural-network-based models show improved fitting accuracy relative to simple regression approaches; however, their performance varies with yarn number and remains sensitive to localized fluctuations in the force signal. Across all multi-yarn configurations, the physics-guided hybrid machine learning (PG-HML) framework consistently demonstrates superior predictive accuracy, as evidenced by the lowest RMSE and highest R^2^ values reported in Table 7. Notably, the performance gap between PG-HML and purely data-driven models becomes more pronounced with increasing yarn numbers. This trend indicates that explicitly embedding stage-wise physical constraints becomes increasingly critical as the mechanical response transitions from single-yarn-dominated behavior to collective load-sharing and friction-controlled regimes. For two-yarn pull-out, the PG-HML framework accurately reproduces the pre-peak stiffness evolution and peak force level while maintaining a smooth and physically admissible post-peak decay. In the three-yarn pull-out configuration, where force magnitudes and interfacial complexity are highest, PG-HML retains stable predictive performance, whereas purely data-driven models exhibit increased scatter and reduced generalization capabilities. The metrics confirm that this robustness is achieved without overfitting, despite the limited experimental dataset. Overall, the multi-yarn results demonstrate that model performance does not scale linearly with increasing mechanical complexity. While conventional machine learning approaches remain adequate for capturing global trends, their predictive reliability deteriorates as inter-yarn interactions intensify. By contrast, the PG-HML framework preserves mechanical continuity and physically meaningful scaling across both two-yarn and three-yarn pull-out configurations, confirming its suitability for predictive modeling of complex textile interphases under friction-dominated loading conditions.

#### 3.4.3. Initial Interlacement Rupture Force–Displacement Modeling and Prediction

The predictive capability of the machine learning models was further assessed for estimating the initial interlacement rupture force, which represents a mechanically critical transition governing load transfer and energy dissipation during yarn pull-out. This parameter is particularly sensitive to inter-yarn friction and nano-functionalization effects, making it a stringent metric for model evaluation. Table 9 summarizes the quantitative prediction performance of the evaluated models, especially for two- and three-yarn pull-out configurations. Across all configurations, conventional regression-based approaches exhibit higher prediction errors, indicating limited robustness in capturing rupture-related force transitions. Tree-based and neural network-based models improve predictive accuracy; however, their performance shows increased variability with yarn number, particularly under three-yarn pull-out conditions where inter-yarn coupling is strongest. Among all evaluated approaches, the physics-guided hybrid machine learning (PG-HML) framework consistently achieves the lowest error levels and highest coefficients of determination for rupture force prediction across all pull-out configurations (Table 9). Importantly, while rupture force prediction becomes increasingly challenging with increasing yarn number, the relative degradation in predictive accuracy is markedly lower for PG-HML compared to purely data-driven models. This behavior confirms that explicit incorporation of physically meaningful constraints enhances model stability for rupture-dominated responses.

The rupture force predictions are governed by the physically consistent formulation defined in Equation (31), which ensures continuity between the pre-rupture and post-rupture regimes.

(31) $$(c_{\mathrm{nh}-B4C},\;n_{y})\;⟶\;\left(F_{\mathrm{rupture}},\;x_{\mathrm{rupture}}\right)$$

where $c_{\mathrm{nh}-B4C}$ denotes the nano hexagonal boron carbide content and $n_{y}$ represents the number of interacting yarns (single-, two-, or three-yarn configurations). By preserving this formulation, the PG-HML framework avoids non-physical force discontinuities and enables reliable scaling of rupture force with nh-B_4_C content and yarn interaction complexity. Overall, the results in Table 8 demonstrate that accurate and robust prediction of initial interlacement rupture force requires more than global curve fitting. While data-driven models capture general trends, the PG-HML framework provides superior mechanical consistency and predictive reliability, particularly for multi-yarn pull-out scenarios where rupture governs subsequent frictional sliding and energy dissipation.

#### 3.4.4. Stick–Slip Regime Learning and Envelope Prediction

The final stage of yarn pull-out is governed by stick–slip behavior, which controls post-rupture frictional sliding and contributes significantly to overall energy dissipation. Unlike pre-peak and rupture stages, the stick–slip regime is inherently stochastic, characterized by local force oscillations superimposed on a decaying force envelope. Consequently, accurate prediction of the global force envelope, rather than exact replication of local fluctuations, constitutes the primary modeling objective in this stage. Table 10 presents a quantitative comparison of model performance for predicting stick–slip behavior across single-yarn, two-yarn, and three-yarn pull-out configurations. Conventional regression-based models exhibit limited accuracy in this regime, reflected by elevated error levels and reduced coefficients of determination. Tree-based and neural-network-based models partially improve predictive performance; however, their accuracy deteriorates with increasing yarn number, indicating sensitivity to amplified frictional interactions and cumulative sliding effects. In contrast, the physics-guided hybrid machine learning (PG-HML) framework consistently demonstrates superior robustness in capturing the stick–slip force envelope, as evidenced by the lowest RMSE and highest R^2^ values reported in Table 10. Importantly, while absolute prediction errors increase for all models with increasing yarn number, the relative degradation in predictive accuracy is substantially lower for PG-HML, confirming its ability to generalize under increasing interfacial complexity.

**Figure 14:**
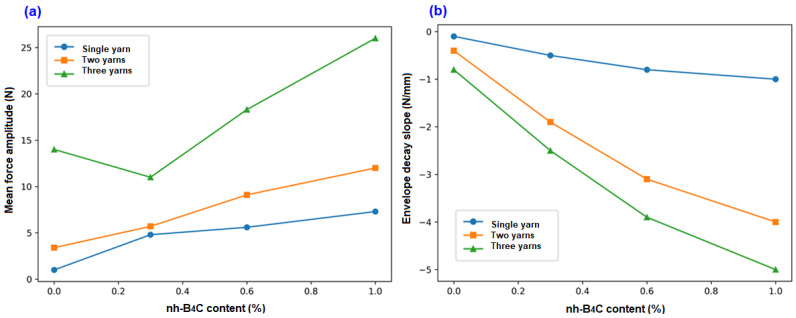
Machine learning-assisted characterization of the macroscopic stick–slip regime in nh-B_4_C-functionalized para-aramid fabrics for different yarn interaction configurations. (**a**) Predicted mean force amplitude as a function of nh-B_4_C content for single-, two-, and three-yarn pull-out, showing a monotonic increase in frictional intensity with both nano-content and interaction density, and (**b**) the predicted friction envelope decay slope, illustrating the systematic steepening of post-peak force decay with increasing yarn multiplicity and nano-functionalization.

The force–displacement responses shown in Figure 14a,b further illustrate these trends. Experimental curves exhibit pronounced local oscillations, whereas the PG-HML predictions suppress noise-sensitive fluctuations and preserve a smooth, physically admissible decay envelope. This behavior reflects the physically informed formulation of the post-rupture regime, ensuring continuity with the preceding rupture stage and preventing non-physical force amplification during sliding. Overall, the combined evidence from Table 10 and Figure 14a,b demonstrates that reliable modeling of stick–slip behavior requires prioritization of envelope fidelity over local oscillation matching. While purely data-driven models capture qualitative trends, the PG-HML framework provides a more consistent and mechanically meaningful representation of post-rupture frictional sliding, making it well-suited for predictive analysis and the parametric exploration of nano-functionalized aramid fabric interfaces.

### 3.5. Nano-Coated Fabric Pull-Out Mechanism

**Figure 15:**
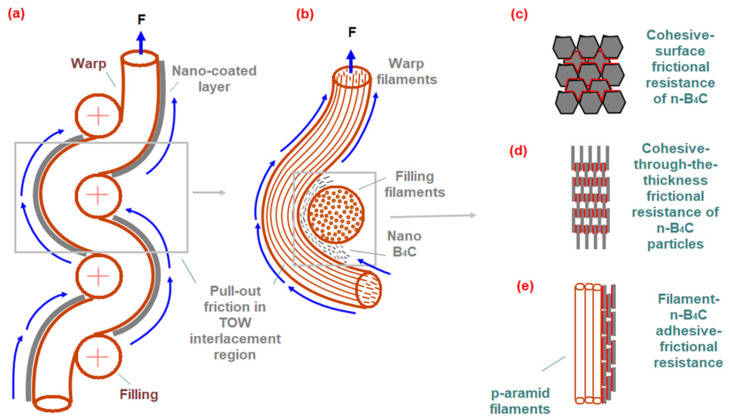
Conceptual illustration of the multiscale pull-out resistance mechanisms in nh-B_4_C-coated p-aramid fabric structures, highlighting (**a**) early-stage yarn extraction [86], (**b**) nano-reinforcement motion within interlacement regions [86], (**c**) inter-particle friction, (**d**) through-thickness interfacial resistance, and (**e**) filament–nh-B_4_C frictional interactions.

Figure 15a–e schematically summarize the multiscale mechanisms governing yarn pull-out resistance in nh-B_4_C-coated para-aramid fabrics, integrating experimental observations with the machine learning-assisted force, shear, and energy responses discussed in previous sections. At the early pull-out stage (Figure 15a), yarn extraction is dominated by crimp extension under the complex geometric constraint imposed by filling yarns. The nh-B_4_C coating increases surface roughness and local contact pressure at warp–filling crossover points, enhancing frictional resistance prior to interlacement rupture. This mechanism is consistent with the elevated crimp extension energy predicted by the ML framework. With increasing displacement (Figure 15b), the nano-coated yarn segment is driven into the interlacement region, where nh-B_4_C particles distributed on filament surfaces and within inter-filament gaps undergo local rearrangement and micro-sliding. This densification of nano-contacts intensifies friction and shear resistance, directly explaining the ML-predicted increases in initial interlacement rupture force and intra-yarn shear strength. A key contribution arises from cohesive inter-particle friction between adjacent nh-B_4_C particles (Figure 15c).

Due to their angular morphology and high surface energy, these particles form displacement-sustained nano–nano contacts that stabilize the force–displacement response after rupture. This mechanism underpins the monotonic increase in stick–slip force amplitude and energy dissipation captured by the ML models. As illustrated in Figure 15d, through-thickness frictional resistance develops as nano-coated filaments interact across adjacent yarn layers during pull-out. This effect becomes increasingly significant with rising yarn interaction density, providing a physical basis for the ML-predicted scaling of shear strength and total energy in multi-yarn configurations. At the filament scale (Figure 15e), nh-B_4_C particles act as discrete adhesive–frictional asperities, promoting intermittent stick–slip sliding. This interaction governs the friction-dominated regime that contributes most strongly to total energy dissipation, in agreement with the ML-predicted stick–slip metrics.

Overall, Figure 15 depicts a hierarchical, friction-dominated pull-out resistance mechanism, in which nanoscale particle interactions, filament-level friction, and yarn-level constraints act synergistically to enhance macroscopic performance. The close agreement between experimental observations and ML-predicted trends confirms that nh-B_4_C functionalization promotes a displacement-sustained, energy-dissipative response rather than a brittle or slip-controlled mechanism, providing a robust physical foundation for the data-driven modeling approach.

## 4. Conclusions

This study developed and validated a stage-wise physics-guided hybrid machine learning (PG-HML) framework for predicting the interfacial yarn pull-out behavior of nano hexagonal boron carbide (nh-B_4_C)-functionalized para-aramid fabrics under single- and multi-yarn extraction conditions. By combining physically meaningful deformation-stage decomposition with supervised learning and physics-informed constraints, the proposed methodology provides a physically interpretable and data-efficient approach for analyzing friction-dominated textile mechanics. The framework successfully reproduced the experimentally measured pull-out responses of both control (KPO, 0 wt.% nh-B_4_C) and nano-functionalized (BPO, 0.3 wt.% nh-B_4_C) fabrics. High predictive accuracy was achieved throughout the critical deformation stages, with coefficient of determination (R^2^) values generally exceeding 0.98 during the crimp extension and rupture regions. The deviations between experimental and predicted peak pull-out forces remained below approximately 1% for single- and three-yarn configurations and within approximately 5% for the two-yarn configuration.

Machine learning predictions demonstrated that increasing nh-B_4_C content significantly enhanced interfacial load transfer and frictional resistance. The maximum pull-out force increased from approximately 25 N to 198 N for single-yarn extraction, from approximately 95 N to 826 N for two-yarn extraction, and from approximately 268 N to 1682 N for three-yarn extraction as nh-B_4_C content increased from 0 to 1.0 wt.%. These findings indicate that the beneficial effects of nano-functionalization become increasingly pronounced as the number of interacting yarns increases.

The results further revealed systematic increases in rupture resistance, intra-yarn shear strength, and pull-out energy dissipation with increasing nano-additive concentration, whereas displacement-related parameters approached saturation, indicating a transition toward friction-controlled pull-out behavior. Comparative evaluation showed that conventional regression, ensemble, kernel-based, and neural-network models were generally effective in the stable pre-peak deformation region but exhibited reduced predictive capability in mechanically critical transition and stick–slip regimes. In contrast, the proposed PG-HML framework maintained superior predictive fidelity by incorporating stage-wise learning strategies and physics-guided continuity constraints.

The principal scientific novelty of this study lies in the development of a stage-wise physics-guided machine learning methodology that explicitly links experimentally observed pull-out mechanisms—crimp extension, initial interlacement rupture, and stick–slip sliding—with data-driven prediction. Unlike conventional black-box machine learning approaches, the proposed framework preserves mechanical interpretability, enforces physically admissible transitions between deformation stages, and achieves reliable predictions under limited data conditions. Consequently, the PG-HML methodology provides an efficient surrogate modeling strategy for the design, optimization, and accelerated development of nano-functionalized soft ballistic textile systems and other friction-dominated fiber-based composite structures.

## Figures and Tables

**Table 1:** Nano-modifier composition and surface coating conditions of the p-aramid fabric/nh-B_4_C samples used in the pull-out experiments.

Sample Code	Structures	Number of Layers	Nano Material and Ratio (%)
KPO	Neat fabric	1	-
BPO	Nano B_4_C coated fabric	1	Nano hexagonal boron carbide (nh-B_4_C, 0.3%)

**Table 2:** Input dataset used for the development, calibration, and validation of the proposed PG-HML framework.

Dataset	Nano Hexagonal Boron Carbide Content (wt.%)	Pull-Out Yarn Configurations	Purpose
KPO (Experimental)	0.0 wt.%	Single yarn	Training/calibration/validation
KPO (Experimental)	0.0 wt.%	Two yarns	Training/calibration/validation
KPO (Experimental)	0.0 wt.%	Three yarns	Training/calibration/validation
BPO (Experimental)	0.3 wt.%	Single yarn	Training/calibration/validation
BPO (Experimental)	0.3 wt.%	Two yarns	Training/calibration/validation
BPO (Experimental)	0.3 wt.%	Three yarns	Training/calibration/validation
PG-HML output	0.1–1.0 wt.%	Single, two, and three yarns	Prediction

**Table 3:** Machine learning-predicted maximum single- and multi-yarn pull-out force–displacement parameters, initial interlacement rupture force, and intra-yarn shear strength of the aramid/B_4_C material.

Label	Pull-Out Force (Max, N)	Displacement (Max, mm)	Initial Interlacement Rupture Force (N)	Initial Interlacement Rupture Displacement (mm)	Actual Initial Interlacement Rupture Displacement (mm)	Intra-Yarn Shear Strength (MPa)	Yarn Tensile Strength (N)
Single yarn							
KPO(0.0%)actual	25.24	6.96	19.20	9.15	2.66	0.0280	493.100 ± 19.78
KPO(0.0%)model	25.07	6.60	22.40	7.94	1.34	0.0275	-
BPO(0.1%)model	45.49	10.72	40.87	15.47	4.75	0.0488	-
BPO(0.2%)model	70.20	10.93	63.15	18.97	8.04	0.0754	-
BPO(0.3%)actual	94.52	11.26	86.47	13.42	2.16	0.1010	460.180 ± 31.09
BPO(0.3%)model	95.04	10.93	85.42	19.39	8.46	0.1020	-
BPO(0.4%)model	120.90	10.93	108.67	19.59	8.66	0.1297	-
BPO(0.5%)model	134.63	10.93	121.13	19.59	8.66	0.1445	-
BPO(0.6%)model	150.91	10.93	134.97	22.89	11.96	0.1619	-
BPO(0.7%)model	167.21	11.03	149.74	22.89	11.86	0.1794	-
BPO(0.8%)model	180.97	11.03	162.19	22.89	11.86	0.1942	-
BPO(0.9%)model	191.00	11.03	171.28	22.89	11.86	0.2049	-
BPO(1.0%)model	197.57	11.03	177.22	22.89	11.86	0.2119	-
Multiple yarns Two yarns							
KPO(0.0%)actual	94.790	7.110	63.370	9.280	2.170	0.052	-
KPO(0.0%)model	92.525	6.600	71.742	6.851	0.251	0.051	-
BPO(0.1%)model	169.802	15.140	169.546	15.479	0.339	0.091	-
BPO(0.2%)model	276.806	15.732	276.369	15.986	0.254	0.149	-
BPO(0.3%)actual	402.420	19.530	377.050	21.940	2.410	0.216	-
BPO(0.3%)model	383.411	16.071	383.400	16.155	0.084	0.206	-
BPO(0.4%)model	495.034	16.409	473.049	16.578	0.169	0.266	-
BPO(0.5%)model	554.284	16.409	529.304	16.578	0.169	0.297	-
BPO(0.6%)model	624.522	16.409	595.991	16.578	0.169	0.335	-
BPO(0.7%)model	694.762	16.409	662.678	16.578	0.169	0.373	-
BPO(0.8%)model	754.012	16.409	718.932	16.578	0.169	0.405	-
BPO(0.9%)model	797.231	16.409	759.966	16.578	0.169	0.428	-
BPO(1.0%)model	825.526	16.409	786.831	16.578	0.169	0.443	-
Three yarns							
KPO(0.0%)actual	267.880	8.140	180.190	8.640	0.500	0.098	-
KPO(0.0%)model	266.998	8.132	197.083	8.599	0.467	0.097	-
BPO(0.1%)model	386.052	14.675	236.550	18.040	3.365	0.138	-
BPO(0.2%)model	590.809	17.573	314.654	18.040	0.467	0.211	-
BPO(0.3%)actual	802.427>	17.562	329.238	18.395	0.833	0.287	-
BPO(0.3%)model	801.621	17.573	392.757	18.040	0.467	0.286	-
BPO(0.4%)model	1021.211	17.573	474.112	18.040	0.467	0.365	-
BPO(0.5%)model	1137.770	17.573	517.296	18.040	0.467	0.407	-
BPO(0.6%)model	1277.916	17.479	568.489	18.040	0.561	0.457	-
BPO(0.7%)model	1419.235	17.479	619.682	18.040	0.561	0.507	-
BPO(0.8%)model	1538.446	17.479	662.866	18.040	0.561	0.550	-
BPO(0.9%)model	1625.401	17.479	694.366	18.040	0.561	0.581	-
BPO(1.0%)model	1682.332	17.479	714.989	18.040	0.561	0.601	-

**Table 4:** Stage-dependent ML-assisted regression models for single- and multi-yarn pull-out force–displacement behavior of nh-B_4_C-functionalized p-aramid substrates.

Label	Equations					
	Crimp Extension Region (Stage I)	Coefficient of Regression (R^2^)	Initial Interlacement Rupture Region (Stage II)	Coefficient of Regression (R^2^)	Stick–Slip Region (Stage III)	Coefficient of Regression (R^2^)
Single yarn						
KPO(0.0%)actual	FI(x) = −0.023x^3^ + 0.114x^2^ + 3.923x + 1.877	0.9887	FII(x) = −0.335x^2^ + 2.644x + 22.504	0.9825	FIII(x) = 28.389 exp (−0.0188x)	0.8995
KPO(0.0%)model	FI(x) = −0.038x^3^ + 0.260x^2^ + 3.512x + 2.165	0.9917	FII(x) = 0.360x^2^ + 2.931x + 21.763	0.9873	FIII(x) = 40.546 exp (−0.0214x)	0.4832
BPO(0.1%)model	FI(x) = −0.033x^3^ + 0.552x^2^ + 1.945x + 1.970	0.9969	FII(x) = 0.481x^2^ − 13.217x + 132.244	0.9886	FIII(x) = 85.269 exp (−0.0202x)	0.7664
BPO(0.2%)model	FI(x) = −0.067x^3^ + 1.517x^2^ − 2.306x + 3.708	0.9967	FII(x) = 0.205x^2^ − 7.609x + 129.205	0.9936	FIII(x) = 132.641 exp (−0.0202x)	0.8001
BPO(0.3%)actual	FI(x) = −0.121x^3^ + 2.793x^2^ − 7.758x + 6.441	0.9937	FII(x) = −0.246x^2^ + 2.077x + 102.390	0.9887	FIII(x) = 156.137 exp (−0.0203x)	0.8962
BPO(0.3%)model	FI(x) = −0.099x^3^ + 2.479x^2^ − 6.543x + 5.436	0.9944	FII(x) = −0.381x^2^5.452x + 81.381	0.9915	FIII(x) = 179.974 exp (−0.0202x)	0.8126
BPO(0.4%)model	FI(x) = −0.134x^3^ + 3.481x^2^ − 10.962x + 7.244	0.9925	FII(x) = −0.992x^2^ + 19.057x + 31.565	0.9902	FIII(x) = 229.374 exp (−0.0202x)	0.8194
BPO(0.5%)model	FI(x) = −0.153x^3^ + 4.035x^2^ − 13.463x + 8.553	0.9917	FII(x) = −1.002x^2^ + 18.774x + 49.765	0.9842	FIII(x) = 255.860 exp (−0.0202x)	0.8223
BPO(0.6%)model	FI(x) = −0.175x^3^ + 4.695x^2^ − 16.542x + 10.561	0.9907	FII(x) = −1.331x^2^ + 26.028x + 26.195	0.9834	FIII(x) = 287.007 exp (−0.0202x)	0.8245
BPO(0.7%)model	FI(x) = −0.210x^3^ + 5.531x^2^ − 20.262x + 13.123	0.9895	FII(x) = −1.491x^2^ + 29.031x + 29.144	0.9831	FIII(x) = 318.162 exp (−0.0202x)	0.8263
BPO(0.8%)model	FI(x) = −0.229x^3^ + 6.080x^2^ − 22.830x + 14.927	0.9887	FII(x) = −1.755x^2^ + 34.801x + 11.437	0.9826	FIII(x) = 344.447 exp (−0.0202x)	0.8274
BPO(0.9%)model	FI(x) = −0.242x^3^ + 6.472x^2^ − 24.669x + 16.237	0.9881	FII(x) = −1.947x^2^ + 39.010x − 1.480	0.9824	FIII(x) = 363.623 exp (−0.0202x)	0.8282
BPO(1.0%)model	FI(x) = −0.250x^3^ + 6.726x^2^ − 25.860x + 17.090	0.9877	FII(x) = −2.073x^2^ + 41.765x − 9.936	0.9822	FIII(x) = 376.178 exp (−0.0202x)	0.8286
Multiple yarns Two yarns						
KPO(0.0%)actual	FI(x) = −0.605x^3^ + 7.091x^2^ − 7.114x + 4.512	0.9962	FII(x) = −11.883x^2^ + 183.033x − 612.221	0.9278	FIII(x) = 85.453 exp (0.0174x)	0.9719
KPO(0.0%)model	FI(x) = −0.617x^3^ + 7.277x^2^ − 8.047x + 5.886	0.9998	FII(x) = 276.367x^2^ − 3803.041x + 13,154.210	0.9783	FIII(x) = 75.238 exp (0.0155x)	0.9928
BPO(0.1%)model	FI(x) = 0.046x^3^ − 2.265x^2^ + 36.488x − 24.032	0.9877	FII(x) = −1.413x^2^ + 42.428x − 148.545	0.9401	FIII(x) = 255.293 exp (0.0139x)	0.9415
BPO(0.2%)model	FI(x) = 0.021x^3^ − 2.001x^2^ + 45.363x − 27.298	0.9967	FII(x) = −0.053x^2^ + 1.087x + 272.424	0.9530	FIII(x) = 437.580 exp (0.0138x)	0.9020
BPO(0.3%)actual	FI(x) = 0.055x^3^ − 3.235x^2^ + 65.133x − 51.769	0.9811	FII(x) = 5.515x^2^ − 242.164x + 3032.557	0.9586	FIII(x) = 655.774 exp (0.0162x)	0.7820
BPO(0.3%)model	FI(x) = −0.003x^3^ − 1.743x^2^ + 54.257x − 30.519	0.9984	FII(x) = −135.950x^2^ + 4409.270x − 35,365.79	0.9297	FIII(x) = 619.987 exp (0.0137x)	0.8818
BPO(0.4%)model	FI(x) = −0.026x^3^ − 1.518x^2^ + 63.761x − 34.134	0.9988	FII(x) = 395.578x^2^ − 13,178.880x + 110,235.807	0.9999	FIII(x) = 811.975 exp (0.0137x)	0.8690
BPO(0.5%)model	FI(x) = −0.040x^3^ − 1.368x^2^ + 68.640x − 35.877	0.9988	FII(x) = 448.44x^2^ − 14,940.225x + 124,964.596	0.9999	FIII(x) = 912.940 exp (0.0137x)	0.8644
BPO(0.6%)model	FI(x) = −0.056x^3^ − 1.190x^2^ + 74.422x − 37.938	0.9987	FII(x) = 511.098x^2^ − 17,028.238x + 14,2425.062	0.9999	FIII(x) = 1032.635 exp (0.0137x)	0.8600
BPO(0.7%)model	FI(x) = −0.072x^3^ − 1.010x^2^ + 80.195x − 39.975	0.9987	FII(x) = 573.759x^2^ − 19,116.250x + 159,885	0.9999	FIII(x) = 1152.332 exp (0.0137x)	0.8564
BPO(0.8%)model	FI(x) = −0.085x^3^ − 0.859x^2^ + 85.064x − 41.693	0.9986	FII(x) = 626.618x^2^ − 20,877.595x + 174,614.318	0.9999	FIII(x) = 1253.304 exp (0.0137x)	0.8539
BPO(0.9%)model	FI(x) = −0.096x^3^ − 0.748x^2^ + 88.617x − 42.947	0.9985	FII(x) = 665.174x^2^ − 22,162.376x + 185,357.962	0.9999	FIII(x) = 1326.957 exp (0.0137x)	0.8523
BPO(1.0%)model	FI(x) = −0.102x^3^ − 0.677x^2^ + 90.942x − 43.767	0.9985	FII(x) = 690.418x^2^ − 23,003.542x + 192,391.998	0.9999	FIII(x) = 1375.179 exp (0.0137x)	0.8513
Three yarns						
KPO(0.0%)actual	FI(x) = −2.415x^3^ + 32.252x^2^ − 74.325x + 27.78	0.9771	FII(x) = −1.928x^2^ + 10.472x + 312.0	0.9999	FIII(x) = 279.294 exp(−0.0381x)	0.7737
KPO(0.0%)model	FI(x) = −1.135x^3^ + 15.867x^2^ − 25.561x + 13.39	0.9891	FII(x) = −563.351x^2^ + 9421.607x − 39,155.1	0.4104	FIII(x) = 244.434 exp(−0.0188x)	0.9553
BPO(0.1%)model	FI(x) = −0.111x^3^ − 0.547x^2^ + 58.751x − 61.47	0.9434	FII(x) = −54.772x^2^ + 1633.944x − 11,809.7	0.3430	FIII(x) = 494.431 exp(−0.0229x)	0.9325
BPO(0.2%)model	FI(x) = −0.012x^3^ − 2.740x^2^ + 88.588x − 96.96	0.9596	FII(x) = 581.510x^2^ − 21,492.139x + 198,833.8	0.9290	FIII(x) = 701.670 exp(−0.0246x)	0.8851
BPO(0.3%)actual	FI(x) = −0.077x^3^ − 1.617x^2^ + 101.126x − 115.36	0.9634	FII(x) = −713.788x^2^ + 24,719.580x − 213,143.3	0.8728	FIII(x) = 891.873 exp(−0.0252x)	0.8842
BPO(0.3%)model	FI(x) = −0.076x^3^ − 1.632x^2^ + 100.961x − 114.19	0.9637	FII(x) = −38.934x^2^ + 602.086x + 2303.4	0.9015	FIII(x) = 869.006 exp(−0.0253x)	0.8438
BPO(0.4%)model	FI(x) = −0.127x^3^ − 0.816x^2^ + 115.811x − 134.39	0.9651	FII(x) = 731.590x^2^ − 27,487.922x + 258,372.8	0.9090	FIII(x) = 1031.394 exp(−0.0259x)	0.8042
BPO(0.5%)model	FI(x) = −0.158x^3^ − 0.294x^2^ + 123.184x − 144.53	0.9654	FII(x) = −371.695x^2^ + 11,837.211x − 91,929.4	0.9203	FIII(x) = 1112.043 exp(−0.0262x)	0.7838
BPO(0.6%)model	FI(x) = −0.204x^3^ + 0.546x^2^ + 130.629x − 155.06	0.9655	FII(x) = −5130.410x^2^ + 181,315.631x − 1,600,689.4	0.9834	FIII(x) = 1202.985 exp(−0.0264x)	0.7605
BPO(0.7%)model	FI(x) = −0.242x^3^ + 1.181x^2^ + 139.271x − 166.97	0.9655	FII(x) = −3989.587x^2^ + 140,786.426x − 1,240,615.6	0.9691	FIII(x) = 1292.202 exp(−0.0266x)	0.7401
BPO(0.8%)model	FI(x) = −0.274x^3^ + 1.716x^2^ + 146.562x − 177.01	0.9655	FII(x) = −1496.304x^2^ + 52,517.018x − 459,279.4	0.9889	FIII(x) = 1366.504 exp(−0.0268x)	0.7248
BPO(0.9%)model	FI(x) = −0.297x^3^ + 2.106x^2^ + 151.879x − 184.33	0.9655	FII(x) = 14.241x^2^ − 848.388x + 12,126.4	0.9999	FIII(x) = 1722.968 exp(−0.0295x)	0.7457
BPO(1.0%)model	FI(x) = −0.312x^3^ + 2.362x^2^ + 155.361x−189.13	0.9654	FII(x) = −11.244x^2^ + 31.294x + 4593.6	0.9999	FIII(x) = 2803.524 exp(−0.0359x)	0.7109

**Table 5:** Quantitative performance evaluation of machine learning-based regression models describing the single-yarn pull-out force–displacement response of nh-B_4_C-functionalized p-aramid substrates.

Label	Mean Square Error (MSE)			Mean Absolute Error (MAE)			Root Mean Square Error (RMSE)			Coefficient of Regression (R^2^)		
	Stage I	Stage II	Stage III	Stage I	Stage II	Stage III	Stage I	Stage II	Stage III	Stage I	Stage II	Stage III
Single yarn												
KPO(0.0%)actual	0.6107	0.0804	3.8440	0.6183	0.2346	1.6671	0.7815	0.2835	1.9606	0.9887	0.9825	0.8995
KPO(0.0%)model	0.4420	0.0523	13.9876	0.5304	0.1903	2.3363	0.6648	0.2287	3.7400	0.9917	0.9873	0.4832
BPO(0.1%)model	0.5885	0.0178	33.4887	0.6406	0.1077	3.4432	0.7671	0.1334	5.7869	0.9969	0.9886	0.7664
BPO(0.2%)model	1.7280	0.0244	72.0322	1.0959	0.1261	4.9296	1.3145	0.1562	8.4872	0.9967	0.9936	0.8001
BPO(0.3%)actual	7.0292	0.0822	103.0431	2.2492	0.2521	7.6170	2.6513	0.2867	10.1510	0.9937	0.9887	0.8962
BPO(0.3%)model	5.8927	0.0697	127.1378	2.0928	0.2089	6.4669	2.4275	0.2640	11.2755	0.9944	0.9915	0.8126
BPO(0.4%)model	13.4300	0.1439	201.6387	3.1825	0.2969	8.0788	3.6647	0.3793	14.1999	0.9925	0.9902	0.8194
BPO(0.5%)model	18.7674	0.3167	247.0401	3.7651	0.4495	8.9206	4.3321	0.5627	15.7175	0.9916	0.9842	0.8223
BPO(0.6%)model	26.7333	0.4331	308.3125	4.5074	0.5235	9.9362	5.1704	0.6581	17.5588	0.9907	0.9834	0.8245
BPO(0.7%)model	38.2448	0.5513	376.4321	5.3840	0.5889	10.9529	6.1842	0.7425	19.4018	0.9895	0.9831	0.8263
BPO(0.8%)model	48.6082	0.6778	439.2177	6.0729	0.6529	11.8115	6.9719	0.8232	20.9575	0.9887	0.9826	0.8274
BPO(0.9%)model	57.1748	0.7783	488.0879	6.5863	0.6995	12.4383	7.5614	0.8822	22.0927	0.9881	0.9824	0.8282
BPO(1.0%)model	63.2462	0.8478	521.4879	6.9272	0.7301	12.8488	7.9527	0.9207	22.8361	0.9877	0.9822	0.8286
Multiple yarns Two yarns												
KPO(0.0%)actual	4.0433	6.5529	12.8819	1.7268	1.7557	2.9155	2.0108	2.5598	3.5891	0.9962	0.9277	0.9719
KPO(0.0%)model	0.1019	1.5631	2.4807	0.1701	1.1182	1.1959	0.3192	1.2502	1.5750	0.9998	0.9783	0.9928
BPO(0.1%)model	38.0464	0.0006	117.4897	4.3475	0.0234	7.3662	6.1682	0.0245	10.8393	0.9877	0.9401	0.9415
BPO(0.2%)model	26.0318	0.0001	530.2162	3.3323	0.0108	15.8634	5.1021	0.0100	23.0264	0.9967	0.9530	0.9020
BPO(0.3%)actual	313.3542	4.3442	2929.0172	14.0378	1.8257	47.4714	17.7018	2.0843	54.1204	0.9811	0.9586	0.7820
BPO(0.3%)model	23.7402	2.7279	1240.7406	2.8468	1.3092	24.3787	4.8724	1.6516	35.2241	0.9984	0.9297	0.8818
BPO(0.4%)model	30.9264	0.0001	2290.6355	3.3220	0.0001	33.1823	5.5612	0.0100	47.8606	0.9988	0.9999	0.8690
BPO(0.5%)model	38.5402	0.0001	2981.3385	4.0290	0.0001	37.8919	6.2081	0.0100	54.6016	0.9988	0.9999	0.8644
BPO(0.6%)model	50.8938	0.0001	3917.9274	4.9476	0.0001	43.4756	7.1339	0.0100	62.5933	0.9987	0.9999	0.8600
BPO(0.7%)model	66.7419	0.0002	4982.3014	5.9064	0.0001	49.0600	8.1696	0.0141	70.5854	0.9987	0.9999	0.8564
BPO(0.8%)model	82.9073	0.0003	5979.5186	6.7347	0.0001	53.7710	9.1053	0.0173	77.3273	0.9986	0.9999	0.8539
BPO(0.9%)model	96.3133	0.0002	6764.2764	7.3420	0.0001	57.2076	9.8139	0.0141	82.2452	0.9985	0.9999	0.8523
BPO(1.0%)model	105.8280	0.0006	7304.2783	7.7459	0.0001	59.4576	10.2873	0.0245	85.4651	0.9985	0.9999	0.8513
Three yarns												
KPO(0.0%)actual	272.5115	0.0001	263.8845	12.2154	0.0001	11.8779	16.5079	0.0100	16.2445	0.9771	0.9999	0.7737
KPO(0.0%)model	80.9315	178.2403	97.3681	6.8123	12.0320	6.4364	8.9962	13.3507	9.8675	0.9891	0.4104	0.9553
BPO(0.1%)model	1076.1237	43.3279	476.9293	27.0252	5.9491	11.5694	32.8043	6.5824	21.8387	0.9434	0.3430	0.9325
BPO(0.2%)model	1714.3916	1229.0735	1612.0187	28.0941	30.3656	22.9282	41.4052	35.0581	40.1499	0.9596	0.9290	0.8851
BPO(0.3%)actual	2856.1869	5704.4081	4191.5389	36.3988	65.5652	43.4228	53.4433	75.5275	64.7421	0.9634	0.8728	0.8842
BPO(0.3%)model	2834.9702	4025.0848	3977.1022	36.1578	54.7060	37.8127	53.2444	63.4435	63.6643	0.9637	0.9015	0.8438
BPO(0.4%)model	4453.8856	6262.8999	8035.3425	46.2256	71.6004	54.3982	66.7374	79.1385	89.6401	0.9651	0.9090	0.8042
BPO(0.5%)model	5499.8552	6857.3366	10,985.6193	51.6425	76.8549	63.6640	74.1610	82.8090	104.8123	0.9654	0.9203	0.7838
BPO(0.6%)model	6916.2404	1042.0317	15,279.1645	58.0858	31.9178	75.0210	83.1639	32.2805	123.6089	0.9655	0.9834	0.7605
BPO(0.7%)model	8510.2675	580.3405	20,349.5927	64.6470	21.4825	86.4029	92.2511	24.0902	142.6520	0.9655	0.9691	0.7400
BPO(0.8%)model	9997.0312	31.4331	25,304.8309	70.2891	5.0146	96.2257	99.9851	5.6055	159.0749	0.9655	0.9889	0.7248
BPO(0.9%)model	11,163.5592	0.0001	26,205.3904	74.4486	0.0001	95.9157	105.6577	0.0100	161.8807	0.9655	0.9999	0.7457
BPO(1.0%)model	11,964.7889	0.0001	32,114.8666	77.1969	0.0001	95.2714	109.3837	0.011	179.2062	0.9654	0.9999	0.7109

**Table 6:** ML-assisted characterization of stick–slip force–displacement parameters for single- and multiple-yarn pull-out behavior in nh-B_4_C-functionalized para-aramid materials.

Label	Start–End Displacement (mm)	Number of Cycles	Amplitude Mean Force (ΔF,N)	Amplitude Max Force (ΔF_max_, N)	Period Mean Length (mm)	Period Max. Length (mm)	Upper Envelope Slop (N/mm)	Lower Envelope Slop (N/mm)	Dominant Frequency (Cycles/mm)	Dominant Wavelength (mm)
Single yarn										
KPO(0.0%)actual	9.49–212.47	97	0.84 ± 0.70	3.22	2.08	3.33	−0.09084	−0.08325	0.00491	203.38
KPO(0.0%)model	6.70–212.44	60	0.68 ± 0.76	3.17	3.34	14.23	−0.09284	−0.08653	0.00485	205.83
BPO(0.1%)model	10.83–212.44	38	1.13 ± 2.18	13.49	4.31	50.42	−0.23946	−0.23889	0.00495	201.70
BPO(0.2%)model	11.03–212.44	19	3.62 ± 5.56	20.58	8.43	64.25	−0.38694	−0.38025	0.00496	201.49
BPO(0.3%)actual	13.42–224.90	98	1.54 ± 1.54	7.25	2.15	4.17	−0.41623	−0.40202	0.00471	211.89
BPO(0.3%)model	11.03–212.44	21	4.81 ± 6.88	30.37	7.76	70.74	−0.51761	−0.50440	0.00496	201.49
BPO(0.4%)model	11.03–212.44	27	5.11 ± 6.54	30.38	6.63	54.96	−0.62399	−0.60632	0.00496	201.49
BPO(0.5%)model	11.03–212.44	29	5.40 ± 7.05	32.92	6.17	52.49	−0.70211	−0.68057	0.00496	201.49
BPO(0.6%)model	11.03–212.44	32	5.59 ± 7.55	35.74	5.59	49.19	−0.79172	−0.76665	0.00496	201.49
BPO(0.7%)model	11.14–212.44	33	6.10 ± 8.37	39.81	5.42	49.19	−0.88271	−0.85336	0.00496	201.39
BPO(0.8%)model	11.14–212.44	33	6.67 ± 9.11	43.25	5.42	49.19	−0.95721	−0.92428	0.00496	201.39
BPO(0.9%)model	11.14–212.44	33	7.10 ± 9.66	45.75	5.42	49.19	−1.01168	−0.97579	0.00496	201.39
BPO(1.0%)model	11.14–212.44	33	7.37 ± 10.01	47.39	5.42	49.19	−1.04729	−1.00964	0.00496	201.39
Multiple yarns Two yarns										
KPO(0.0%)actual	6.78–210.43	115	3.32 ± 1.70	30.08	1.76	3.33	−0.33426	−0.31504	0.00490	204.05
KPO(0.0%)model	6.68–210.36	88	0.90 ± 0.07	35.49	2.29	114.10	−0.32826	−0.32608	0.00491	203.72
BPO(0.1%)model	15.23–210.36	75	2.36 ± 0.19	75.19	2.59	83.31	−0.83600	−0.83830	0.00512	195.18
BPO(0.2%)model	15.82–210.36	73	4.13 ± 0.39	116.95	2.65	79.93	−1.36484	−1.36663	0.00514	194.59
BPO(0.3%)actual	16.53–227.18	94	7.12 ± 2.68	37.46	2.22	20.83	−1.75883	−1.75468	0.00474	211.06
BPO(0.3%)model	16.16–210.36	75	5.70 ± 0.62	160.90	2.58	80.02	−1.90203	−1.89990	0.00515	194.25
BPO(0.4%)model	16.49–210.36	72	7.47 ± 0.83	206.68	2.68	80.02	−2.43604	−2.43728	0.00516	193.91
BPO(0.5%)model	16.49–210.36	74	8.17 ± 0.96	230.98	2.61	80.02	−2.73152	−2.73215	0.00516	193.91
BPO(0.6%)model	16.49–210.36	75	9.16 ± 1.16	259.79	2.57	80.02	−3.07721	−3.07795	0.00516	193.91
BPO(0.7%)model	16.49–210.36	75	10.23 ± 1.31	288.60	2.57	80.02	−3.42225	−3.42278	0.00516	193.91
BPO(0.8%)model	16.49–210.36	76	10.98 ± 1.45	312.90	2.54	80.02	−3.71281	−3.71362	0.00516	193.91
BPO(0.9%)model	16.49–210.36	76	11.63 ± 1.55	330.63	2.54	80.02	−3.92512	−3.92581	0.00516	193.91
BPO(1.0%)model	16.49–210.36	76	12.06 ± 1.53	342.23	2.54	80.02	−4.06412	−4.06479	0.00516	193.91
Three yarns										
KPO(0.0%)actual	8.13–210.22	107	14.17	45.82	1.75	5.89	−0.75600	−0.75600	0.004950	202.18
KPO(0.0%)model	8.13–210.22	107	14.16	45.82	1.74	5.88	−0.75583	−0.75583	0.004946	202.1799
BPO(0.1%)model	14.68–210.22	94	7.76	25.73	1.75	7.47	−1.35333	−1.35333	0.005112	195.6369
BPO(0.2%)model	17.57–210.22	80	7.03	40.44	1.58	10.28	−1.88371	−1.88371	0.005188	192.7393
BPO(0.3%)actual	8.13–210.22	88	2.66	4.51	1.66	15.80	−0.74600	−0.74600	0.004950	202.18
BPO(0.3%)model	17.57–210.22	62	11.09	148.29	1.34	12.61	−2.48795	−2.48795	0.005188	192.7393
BPO(0.4%)model	17.57–210.22	76	13.81	111.51	1.48	9.16	−3.11734	−3.11734	0.005188	192.7393
BPO(0.5%)model	17.57–210.22	82	15.86	130.79	1.65	9.72	−3.45142	−3.45142	0.005188	192.7393
BPO(0.6%)model	17.48–210.22	83	18.54	120.67	1.63	9.81	−3.85824	−3.85824	0.005186	192.8327
BPO(0.7%)model	17.48–210.22	86	21.02	139.10	1.64	8.60	−4.25557	−4.25557	0.005186	192.8327
BPO(0.8%)model	17.48–210.22	89	22.68	125.31	1.58	8.60	−4.59073	−4.59073	0.005186	192.8327
BPO(0.9%)model	17.48–210.22	90	24.15	135.58	1.56	8.60	−4.83521	−4.83521	0.005186	192.8327
BPO(1.0%)model	17.48–210.22	88	25.86	142.31	1.60	8.60	−4.99527	−4.99527	0.005186	192.8327

**Table 7:** Single- and multi-yarn pull-out energy values obtained for para-aramid fabric structures incorporating different nh-B_4_C coating contents.

Label	Crimp Extension Energy (J)	Initial Interlacement Rupture Energy (J)	Stick–Slip Energy (J)	Total Energy (J)
Single yarn				
KPO(0.0%)actual	0.095	0.059	1.342	1.496
KPO(0.0%)model	0.098	0.032	1.366	1.497
BPO(0.1%)model	0.249	0.202	2.801	3.253
BPO(0.2%)model	0.326	0.523	4.160	5.009
BPO(0.3%)actual	0.425	0.195	6.152	6.772
BPO(0.3%)model	0.393	0.747	5.624	6.765
BPO(0.4%)model	0.464	0.975	7.156	8.594
BPO(0.5%)model	0.501	1.086	7.978	9.566
BPO(0.6%)model	0.547	1.676	8.496	10.719
BPO(0.7%)model	0.610	1.841	9.420	11.872
BPO(0.8%)model	0.651	1.994	10.200	12.845
BPO(0.9%)model	0.680	2.105	10.769	13.554
BPO(1.0%)model	0.699	2.178	11.141	14.019
Multiple yarns Two yarns				
KPO(0.0%)actual	0.268	0.230	4.196	4.695
KPO(0.0%)model	0.268	0.337	3.971	4.576
BPO(0.1%)model	1.796	0.762	12.708	15.266
BPO(0.2%)model	2.911	1.273	21.775	25.959
BPO(0.3%)actual	4.166	1.934	31.049	37.149
BPO(0.3%)model	4.055	1.815	30.781	36.652
BPO(0.4%)model	5.317	2.252	40.220	47.790
BPO(0.5%)model	5.918	2.521	45.263	53.702
BPO(0.6%)model	6.631	2.839	51.241	60.711
BPO(0.8%)model	7.945	3.426	62.261	73.632
BPO(0.9%)model	8.383	3.622	65.939	77.945
BPO(1.0%)model	8.670	3.750	68.348	80.768
Three yarns				
KPO(0.0%)actual	0.868	0.115	10.352	11.335
KPO(0.0%)model	0.866	0.109	10.361	11.336
BPO(0.1%)model	3.560	1.214	14.041	18.816
BPO(0.2%)model	6.719	0.243	19.334	26.296
BPO(0.3%)actual	8.813	0.457	24.642	33.913
BPO(0.3%)model	8.822	0.326	24.627	33.776
BPO(0.4%)model	11.014	0.413	30.140	41.567
BPO(0.5%)model	12.177	0.459	33.067	45.703
BPO(0.6%)model	13.436	0.634	36.536	50.606
BPO(0.7%)model	14.802	0.701	40.005	55.508
BPO(0.8%)model	15.954	0.758	42.932	59.644
BPO(0.9%)model	16.794	0.800	45.067	62.661
BPO(1.0%)model	17.345	0.827	46.464	64.636

**Table 8:** Quantitative comparison of machine learning model generalization under increasing yarn interaction complexity. Average prediction performance (RMSE and R^2^) for single-, two-, and three-yarn pull-out configurations, evaluated over the full nh-B_4_C content range.

Model	RMSE (Single Yarn)	RMSE (Two Yarns)	RMSE (Three Yarns)	R^2^ (Single Yarn)	R^2^ (Two Yarns)	R^2^ (Three Yarns)
Linear Regression	18.20	41.60	79.40	0.91	0.72	0.48
Polynomial Regression	6.50	14.20	28.70	0.99	0.94	0.81
Ridge Regression	7.30	16.10	31.90	0.98	0.93	0.79
Lasso Regression	7.80	17.40	34.60	0.98	0.92	0.76
Support Vector Regression (RBF)	7.90	18.60	35.20	0.97	0.91	0.75
Random Forest	5.40	12.10	23.80	0.995	0.96	0.86
Gradient Boosting	5.10	11.60	22.90	0.996	0.96	0.87
XGBoost	4.80	11.20	21.70	0.996	0.97	0.88
Neural Network (MLP)	7.80	18.40	35.60	0.970	0.90	0.73
PG-HML (proposed)	4.30	8.90	16.50	0.997	0.98	0.92

**Table 9:** Quantitative performance comparison of machine learning models for multi-yarn pull-out force–displacement prediction.

Yarn Configuration	ML Model	MAE (N)	RMSE (N)	R^2^ (Overall)	R^2^ (Stage I)	R^2^ (Stage II)	R^2^ (Stage III)
Two yarns	Linear Regression	18.42	26.91	0.931	0.982	0.941	0.861
	Ridge Regression	17.96	26.11	0.936	0.984	0.948	0.868
	Lasso Regression	18.77	27.54	0.927	0.981	0.939	0.852
	Polynomial Regression (deg = 3)	11.83	17.92	0.971	0.995	0.983	0.901
	SVR (RBF kernel)	14.26	21.45	0.958	0.989	0.972	0.887
	Random Forest	9.87	15.21	0.981	0.998	0.991	0.912
	Gradient Boosting	9.12	14.38	0.984	0.998	0.994	0.918
	XGBoost	8.76	13.94	0.986	0.999	0.996	0.921
	Neural Network (MLP)	13.94	22.67	0.952	0.991	0.966	0.841
	PG-HML (proposed)	8.21	13.02	0.989	0.999	0.997	0.926
Three yarns	Linear Regression	36.85	54.72	0.882	0.961	0.903	0.792
	Ridge Regression	35.92	53.31	0.889	0.964	0.911	0.801
	Lasso Regression	37.64	56.48	0.874	0.958	0.898	0.781
	Polynomial Regression (deg = 3)	25.17	38.94	0.926	0.987	0.948	0.832
	SVR (RBF kernel)	29.83	44.26	0.908	0.981	0.936	0.819
	Random Forest	21.46	32.78	0.942	0.993	0.966	0.861
	Gradient Boosting	20.18	31.04	0.948	0.994	0.971	0.867
	XGBoost	19.44	29.87	0.952	0.995	0.975	0.871
	Neural Network (MLP)	30.91	47.83	0.901	0.979	0.921	0.803
	PG-HML (proposed)	18.72	28.41	0.956	0.996	0.978	0.879

Error metrics are averaged over all nh-B_4_C contents.

**Table 10:** Machine learning-predicted macroscopic stick–slip descriptors for para-aramid fabrics with varying nh-B_4_C contents under single-, two-, and three-yarn pull-out configurations. The reported parameters characterize the global friction envelope rather than individual micro-oscillations.

Yarn Configuration	nh-B_4_C Content (%)	Mean Force Amplitude (ΔF, N)	Max Force Amplitude (ΔF_max_, N)	Envelope Decay Slope (N/mm)	Cycle Density (Cycles/mm)
Single yarn	0.0	0.84	3.22	−0.091	0.0049
	0.3	4.81	30.37	−0.518	0.0050
	0.6	5.59	35.74	−0.792	0.0050
	1.0	7.37	47.39	−1.047	0.0050
Two yarns	0.0	3.32	30.08	−0.334	0.0049
	0.3	5.70	160.90	−1.902	0.0051
	0.6	9.16	259.79	−3.077	0.0052
	1.0	12.06	342.23	−4.064	0.0052
Three yarns	0.0	14.17	45.82	−0.756	0.0050
	0.3	11.09	148.29	−2.488	0.0052
	0.6	18.54	120.67	−3.858	0.0052
	1.0	25.86	142.31	−4.995	0.0052

## Data Availability

The data supporting the findings of this study are included within this article. The raw experimental pull-out force–displacement datasets used for training, calibration, and validation of the proposed Physics-Guided Hybrid Machine Learning (PG-HML) framework are available from the corresponding author upon reasonable request for the purpose of scientific research and verification.
